# Lessons from discovery of true ADAR RNA editing sites in a human cell line

**DOI:** 10.1186/s12915-023-01651-w

**Published:** 2023-07-19

**Authors:** Fang Wang, Huifen Cao, Qiu Xia, Ziheng Liu, Ming Wang, Fan Gao, Dongyang Xu, Bolin Deng, Yong Diao, Philipp Kapranov

**Affiliations:** 1 Institute of Genomics, School of Medicine, Huaqiao University, 668 Jimei Road, Xiamen, 361021 China; 2 State Key Laboratory of Cellular Stress Biology, School of Life Sciences, Xiamen University, Xiamen, 361102 China

**Keywords:** RNA editing, ADAR, lncRNA, vlincRNA, Cancer, RNA dark matter

## Abstract

**Background:**

Conversion or editing of adenosine (A) into inosine (I) catalyzed by specialized cellular enzymes represents one of the most common post-transcriptional RNA modifications with emerging connection to disease. A-to-I conversions can happen at specific sites and lead to increase in proteome diversity and changes in RNA stability, splicing, and regulation. Such sites can be detected as adenine-to-guanine sequence changes by next-generation RNA sequencing which resulted in millions reported sites from multiple genome-wide surveys. Nonetheless, the lack of extensive independent validation in such endeavors, which is critical considering the relatively high error rate of next-generation sequencing, leads to lingering questions about the validity of the current compendiums of the editing sites and conclusions based on them.

**Results:**

Strikingly, we found that the current analytical methods suffer from very high false positive rates and that a significant fraction of sites in the public databases cannot be validated. In this work, we present potential solutions to these problems and provide a comprehensive and extensively validated list of A-to-I editing sites in a human cancer cell line. Our findings demonstrate that most of true A-to-I editing sites in a human cancer cell line are located in the non-coding transcripts, the so-called RNA 'dark matter'. On the other hand, many ADAR editing events occurring in exons of human protein-coding mRNAs, including those that can recode the transcriptome, represent false positives and need to be interpreted with caution. Nonetheless, yet undiscovered authentic ADAR sites that increase the diversity of human proteome exist and warrant further identification.

**Conclusions:**

Accurate identification of human ADAR sites remains a challenging problem, particularly for the sites in exons of protein-coding mRNAs. As a result, genome-wide surveys of ADAR editome must still be accompanied by extensive Sanger validation efforts. However, given the vast number of unknown human ADAR sites, there is a need for further developments of the analytical techniques, potentially those that are based on deep learning solutions, in order to provide a quick and reliable identification of the editome in any sample.

**Supplementary Information:**

The online version contains supplementary material available at 10.1186/s12915-023-01651-w.

## Background

RNA editing refers to a suite of epitranscriptomic molecular phenomena that result in changes to sequences of specific RNA molecules via insertion, deletion, or substitution of nucleotides at specific positions [1]. A subtype of RNA editing most relevant to mammalian systems is deamination of A into I catalyzed by enzymes belonging to the ADAR (adenosine deaminases acting on RNA) family [2]. The inosines in RNAs are recognized primarily as guanosines (G) inside a cell [3, 4] and also by DNA polymerases used in various sequencing technologies [5].

Mammalian A-to-I type of RNA editing has two major well-characterized physiological functions mediated by two ADARs: ADAR1 and ADAR2 [2, 6]. First, ADAR1-mediated editing of double-stranded (ds) RNAs formed by annealing of sequences corresponding to ubiquitous repeated elements in mammalian genomes attenuates innate immunogenic response caused by these dsRNAs [6, 7]. In fact, the vast majority of human A-to-I editing sites are located within the Alu family of repetitive elements, primarily in the non-coding parts of the genome [8]. Second, ADAR2-mediated editing of GRIA2 mRNA encoding glutamate ionotropic receptor AMPA type subunit 2 leads to an amino acid change in the protein product of the edited transcript that is critical for viability [9, 10]. In addition to the above two functions, A-to-I editing has been implicated in regulation of splicing [11], miRNA target specificity [12–14], and mRNA stability [15, 16]. Furthermore, in addition to GRIA2, other well-characterized A-to-I RNA editing events have been shown to recode amino acid sequences of other mammalian proteins, such as serotonin receptor 5-HT_2C_R [17] and GLI1 transcription factor [18].

All in all, this type of post-transcriptional RNA modification has attracted a significant amount of research interest, including multiple genome-wide studies focused on mapping ADAR editing events and measuring editing levels across multiple cell types and species. The advent of next-generation sequencing (NGS) has caused an explosion of the A-to-I editing sites with millions identified the in the human genome [8, 19–24] using a slew of analytical techniques [25–30]. However, despite the great interest towards this phenomenon, the field of ADAR RNA editing has two issues: biological and technological. First, double ADAR1 and ADAR2 knockout mice, made in a genetic background that can bypass the effect of editing on the innate immunity and GRIA2 and exhibiting no detectable A-to-I editing, are perfectly viable, healthy, and have no obvious phenotypes [31, 32]. Thus, biological significance of the multitude of ADAR editing events outside of those involved in the attenuation of innate immune response and GRIA2 recoding for the normal homeostasis in mammals is questionable. On the other hand, the relationship between ADAR RNA editing and disease in humans, particularly cancer, represents an actively developing area of research [33–36]. ADAR editing in cancer cells have been shown to associate with patients’ survival [34], affect cancer cell viability [33, 35], and increase the diversity of cancer cell proteome [36]. Second, only a tiny fraction of editing sites discovered by NGS and reported in multiple studies have been independently validated by the highly accurate Sanger sequencing. However, such validation is critical since NGS methods have high error rate, which can result in many false positive RNA editing sites as shown by the study of St. Laurent et al. [37]. Therefore, the accuracy of the published RNA detection methods and editing sites detected using them is not clear.

**Fig. 1:**
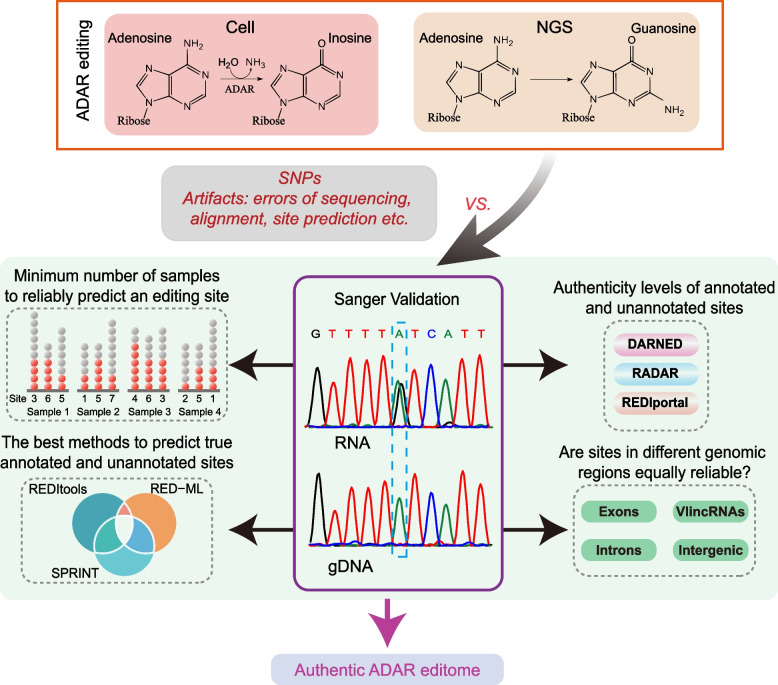
A scheme illustrating the underlying concept of this study. True A-to-I RNA editing events revealed by A-to-G substitutions during RNA sequencing have to be separated from other A-to-G sequence changes that are unrelated to ADAR editing. This work is based on extensive independent validation by Sanger sequencing as to key component to test every indicated step of the analytical pipeline in order to provide an authentic compendium of true ADAR editing events in a particular biological system

Therefore, based on these considerations, in this study, we have performed a genomic survey of ADAR editome that was different from most previous endeavors in two major ways. First, instead of generating a wide survey of editing sites in many different cell or tissue types, we focused on generating as comprehensive collection of ADAR event in a single cell type as possible. Second, and more importantly, we made authenticity of the detected editing sites the top priority in this study by performing extensive Sanger validation of the candidate sites (Fig. 1). We have chosen a well-studied human leukemia cell line K562 that is also a Tier I cell line for the ENCODE consortium [38] as a proxy for an average human cancer cell line system. By performing editing site prediction on 130 different RNA-seq samples from this cell line, we detected ~200 thousand candidate ADAR sites in the non-repeat portion of the genome which we further refined to 3160 annotated and 989 unannotated high confidence sites. Strikingly, we found that detection of true RNA editing sites in NGS data is still a very complex task due to a very high fraction of false positives found even in annotated editing sites deposited in the existing databases and especially among the unannotated sites. Interestingly, the fraction of true RNA editing events depended on (1) the analytical method used, even though none of the tested methods was perfect, and (2) genomic context, with most of the predicted sites in the protein-coding exons found to be false positives and most of the real editing events were located in non-coding transcripts. Overall, our results suggest that conclusions of genomic editing surveys, especially those based on sites located in protein-coding regions, in mammalian systems have to be interpreted with great caution and supported by extensive Sanger validation. We also found that the non-coding transcriptome represents the major reservoir of true ADAR editing events in a human cancer cell. Finally, we provide a pipeline that could be used to generate authentic editome of a mammalian cell.

## Results

### Prediction of ADAR editing sites in a human cancer cell line

ADAR-mediated editing of the same transcript can be influenced by a cell type [39], at least in part, due to influence of various RNA binding proteins [40] and small RNAs [41] as well as levels of various ADAR enzymes [42] (reviewed in [43]). Therefore, to ensure comprehensive detection of ADAR RNA editing events, we analyzed 130 RNA-seq samples comprising 114 samples where K562 cells were treated with different anticancer drugs for variable periods of time and 16 samples representing K562 cells stably transduced with different lentiviral vectors (Additional file 1: Supplementary Tables 1 and 2). The RNA-seq samples used in this study consisted of 34 samples generated by our group in previous publications [44, 45], and 96 samples generated in this study and are listed together with the corresponding GEO accession numbers in the Additional file 1: Supplementary Table 1. Based on our previous work, we found that treatment of K562 cell lines with anticancer drugs used in this study can cause significant perturbations in the expression levels of both protein-coding and non-coding transcriptomes [45]. While it is hard to predict how any individual treatment would impact editing levels of specific sites, the multitude of different treatments used in this study should, in theory, provide a sufficiently diverse states of the transcriptome to permit detection of as many sites as could be reasonably expected for a single cell type. Still, it is important to emphasize that this work is limited to only one cell type.

In each sample, RNA-seq analysis was performed on total RNA containing both polyA+ and polyA− fractions to ensure detection of ADAR editing events in the non-coding transcriptome that tends to be non-polyadenylated [46]. Overall, 17,981,458–49,289,296 quality-filtered 150 bp paired-end Illumina reads were obtained from each sample, constituting a total of 4,831,775,274 reads (Additional file 1: Supplementary Table 1). RNA editing events were then predicted in each sample by each of the three different analytical tools: RED-ML [27], REDItools [25], and SPRINT [26] (Fig. 1, Additional file 2: Supplementary Figure 1, Methods). These tools were chosen because they are downloadable as standalone applications and have been used widely by the community to predict RNA editing events based on the NGS data. For example, REDItools has been used to predict millions of ADAR editing sites from thousands of RNA-seq experiments [23, 24]. Then, all candidate editing sites found by each method in each sample were filtered to remove sequence variants present in human dbSNP v151 or found by in-house resequencing of the K562 genome. Overall, REDItools, RED-ML, and SPRINT detected 1,632,062, 1,028,607, and 2,944,010 ADAR editing sites, respectively.

**Fig. 2:**
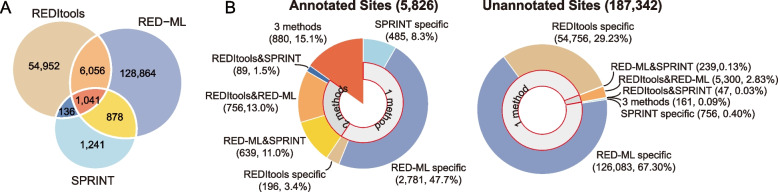
Performance of the different analytical techniques in ADAR site detection. **a** A Venn diagram showing the number of the candidate editing sites predicted by one or multiple methods. Only sites with the maximum editing level of > 0.2 across all 130 samples were used in this analysis. **b** The numbers and fractions of the candidate annotated (left) and unannotated sites (right) editing sites detected by only one (REDItools-, RED-ML-, or SPRINT-specific) or multiple different methods. Source data are provided as a Source data file

As expected, a significant fraction (76.5%) of the total 5,604,679 sites detected by at least one method mapped to repeats of which majority (80.5%) were located in the Alu repeats (Additional file 1: Supplementary Table 3). The sites mapping to repeats were removed from the subsequent analyses since they likely represent the pervasive editing events involved in preventing the dsRNA-mediated innate immune response. The remaining candidate sites were further filtered for potential artifacts of mis-alignments by removing the ones mapping to genomic regions with low sequence uniqueness, resulting in 1,069,339, 152,601, and 19,130 sites predicted by REDItools, RED-ML, and SPRINT, respectively (Methods). Then, we further filtered the candidate sites to only keep those with editing levels > 0.2 since such sites are more likely to have physiological effect, resulting in respectively 62,185, 136,839, and 3296 sites that represented 193,168 unique candidate sites and were used for the downstream analyses (Fig. 2a, Additional file 1: Supplementary Table 4, Methods).

As the first natural step in the analysis, we compared our datasets with thousands of editing sites already annotated in the public databases (Fig. 1, Additional file 2: Supplementary Figure 1). For this purpose, we used DARNED [21], RADAR [20], and REDIportal v2.0 [22] databases containing respectively 8202, 52,494 and 455,619 non-repeat human editing sites corresponding to a total of 457,808 unique sites that will be referred to as the “annotated sites” below. Only 5826 or 3% of the 193,168 candidate sites predicted by our pipeline corresponded to the annotated sites (Fig. 2b, Additional file 1: Supplementary Table 4). Interestingly, most of the annotated sites (59.4%, 3462/5826) identified in our survey were found by only one method, while only 25.5% (1484/5826) and 15.1% (880/5826) were predicted by either two or all three methods (Fig. 2b, Additional file 1: Supplementary Table 4). Strikingly, however, the fraction of unannotated sites detected by one method was much higher (96.9% or 181,595 out of 187,342) while merely 3% (5586/187,342) and 0.1% (161/187,342) of the unannotated sites were detected by two or all three methods, respectively (Fig. 2b, Additional file 1: Supplementary Table 4).

**Fig. 3:**
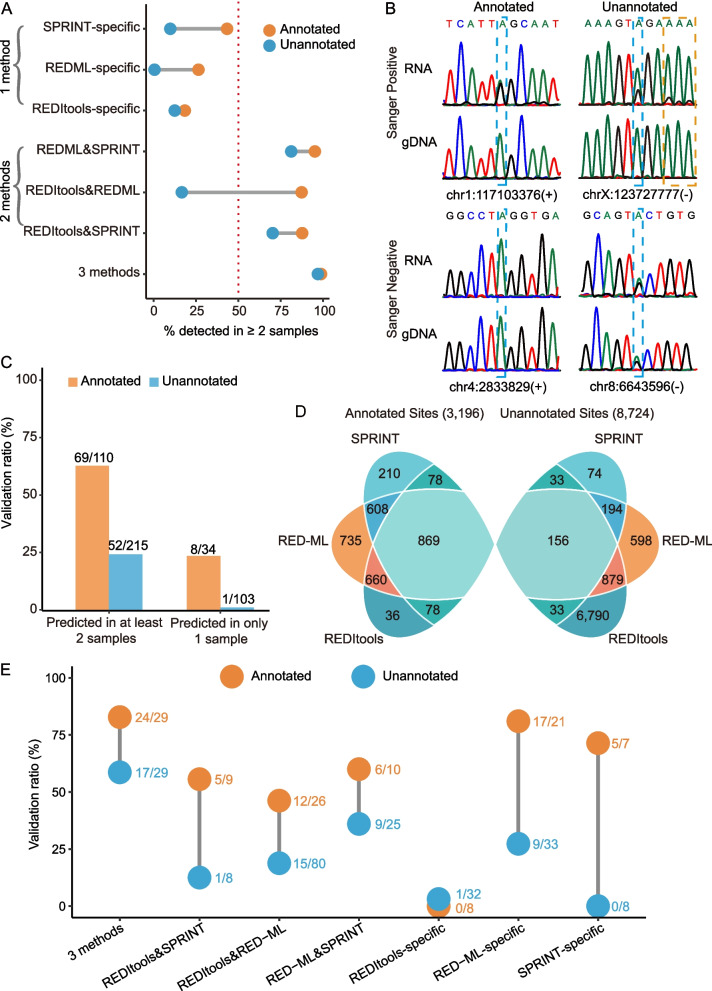
True ADAR editing sites are reproducible in independent RNA-seq samples of the same cell type. **a** Fractions of candidate sites detected in at least two samples (*X*-axis) by only one (REDItools-, RED-ML- or SPRINT-specific), or multiple different methods are shown for annotated (orange circles) and unannotated (blue circles) sites. **b** Examples of typical Sanger validation results for 2 true (top) and 2 false (bottom) ADAR editing sites. Sanger sequencing electropherograms derived from RNA or genomic DNA (gDNA) are shown. The sites targeted for validation are demarcated by the blue dashed lines and their genomic coordinates are given below. The nearby ADAR sites found by Sanger only are demarcated by the orange dashed lines. Note that the false positive site on the bottom right represents an SNP in DNA that was absent from the SNP databases and missed by the K562 genomic resequencing. **c** Validation ratios (*Y*-axis) of the annotated (orange) and unannotated (blue) sites predicted in only one or at least two samples. **d** A Venn diagram representing the number of annotated (left) and unannotated sites (right) which were detected by one or more different methods in at least two samples. **e** Validation ratios (*Y*-axis) of annotated (orange circles) and unannotated (blue circles) sites detected in at least two samples by only one or multiple different methods. **a**–**e** Only sites with the maximum editing level of > 0.2 across all 130 samples were used in this analysis. Source data are provided as a Source data file

The annotated and unannotated sites also differed significantly in terms of the number of samples in which they were found — while 45.1% (2630/5826) of the annotated sites were detected in only 1 of the 130 samples, this ratio increased to 95.3% in the unannotated sites (Additional file 1: Supplementary Table 5). Furthermore, we also found that majority of sites found in only one sample were detected by only one analytical method, while the sites predicted in at least two samples had a tendency to be found by at least two methods (Fig. 3a, Additional file 1: Supplementary Table 5). Since the lack of consistent detection could indicate false positive signal, we therefore first tested whether candidate sites detected in just one sample could be validated using Sanger sequencing (Fig. 1). All sites subjected to Sanger validation were first tested for the evidence of editing in the same RNA preparation used for RNA-seq experiments in which these sites were originally detected. Then, the positive sites were further tested on K562 genomic DNA to exclude the possibility of artificial editing sites caused by DNA sequence variants as illustrated in Fig. 3b (Methods, Additional file 1: Supplementary Tables 7 and 8, see Additional file 3: Supplementary Figure 2 for summary of all sites subjected to Sanger validation and Additional file 4: Supplementary Figure 3, Additional file 5: Supplementary Figure 4 for electropherograms of all sites tested by Sanger sequencing in this study).

Strikingly, only 1% (1/103) and 23.5% (8/34) of the respectively unannotated and annotated sites detected in just one sample could be validated (Fig. 3c, Additional file 1: Supplementary Table 6). Considering that the unannotated sites significantly outnumbered the annotated ones, the actual weighted average of the validation ratio of all sites detected in at least one sample was only 1.3%. On the other hand, the corresponding validation ratio increased to 34.5% for the 325 tested sites predicted in at least two samples based on 62.7% (69/110) and 24.2% (52/215) validation ratios for the annotated and unannotated sites (Fig. 3c). It is important to note that a site was always tested by Sanger sequencing in just one RNA sample where it was originally found, irrespective of whether it was detected in only that sample or in some other sample(s). In other words, all tested sites had the same chance of being detected by the Sanger sequencing, which is an important consideration since it is likely that the sensitivity of Sanger validation is less than 100% and some true sites would be missed. However, based on the experimental design, there is no reason to expect that the sensitivity would have been different for the sites detected in just one or multiple samples if they both contained equal fraction of true positives. Taken together, these results suggested that sites predicted in only 1 sample had much higher chances of being false positives than sites found in 2 or more samples, which is especially true for the unannotated sites. Therefore, we further filtered the candidate editing sites to 3196 annotated and 8724 unannotated sites predicted in at least two samples by at least one analytical method which represented respectively 54.9% and 4.7% of all initial sites (Fig. 3c, Additional file 1: Supplementary Tables 5 and 7-8).

The inability to accurately predict editing sites in a single RNA-seq experiment also suggested that the existing analytical tools need significant improvements and that sites generated by them need to be further filtered and validated. Unfortunately, despite having a large collection of different biological conditions of the same cell type, this limitation has prevented us from determining sample-specific editing sites (e.g., editing sites induced by treatment with a particular anticancer drug). Instead, we used the large number of independent samples to obtain as complete and authentic of a compendium of ADAR editing sites in a cancer cell line as possible. Furthermore, in addition to the failure to predict authentic editing sites from individual samples, there was little overlap among the 3 analytical techniques: as shown in the Fig. 3d, only 27.2% (869/3196) and 1.8% (156/8724) of respectively annotated and unannotated sites were detected by all 3 analytical methods (Additional file 1: Supplementary Table 9). Therefore, as the next step, we explored performance of the different analytical tools based on the Sanger validation.

### Large differences in performance of editing site detection among different analytical techniques

In terms of the annotated editing sites, the three analytical tools differed mostly in terms of the sensitivity, but less so in the accuracy. Of the 3196 unique annotated detected sites, 1643 (51.4%), 1765 (55.2%), and 2872 (89.9%) were found by respectively REDItools, SPRINT, and RED-ML (Fig. 3d, Additional file 1: Supplementary Tables 7-9). The corresponding validation ratios were 64.9%, 72.4%, and 69.1% (Additional file 1: Supplementary Tables 7-9). Considering both sensitivity and accuracy, RED-ML performed significantly better than the other two methods. For example, RED-ML could detect much more unique sites (735) compared to 210 sites found by SPRINT and 36 sites by REDItools (Fig. 3d, Additional file 1: Supplementary Table 9). However, the validation ratio of the sites unique to RED-ML was also quite high (81% or 17/21), compared to 71.4% (5/7) and 0% (0/8) for the sites detected only by SPRINT or REDItools (Fig. 3e, Additional file 1: Supplementary Table 9). Interestingly, increasing the number of methods required to detect each site did not significantly improve the overall performance in the case of the annotated sites. For example, the validation ratio of the sites detected by all three methods was only 82.8% (24/29), and it came with the cost of losing > 70% of all sites, while the validation ratio of sites found by both RED-ML and SPRINT (60.0% or 6/10) was similar to those of the sites detected by each method alone (Fig. 3e, Additional file 1: Supplementary Table 9). Therefore, in terms of an editome survey limited to the annotated sites, RED-ML alone (or in union with SPRINT to slightly improve the sensitivity) can provide adequate results if more than one RNA-seq sample is available. However, still, a large fraction of the detected sites could be false indicative of a relatively high false positive ratios in the current databases (see below).

However, the situation was markedly different in the case of unannotated editing site discovery. First, the outputs of the methods showed far greater variation in terms of both the sensitivity and the accuracy. Of the 8724 unique unannotated detected sites, 7858 (90.1%), 457 (5.2%), and 1827 (20.9%) were found by respectively REDItools, SPRINT, and RED-ML (Fig. 3d, Additional file 1: Supplementary Table 9). Second, independent detection of sites by multiple methods could significantly improve the accuracy. For example, we found the highest validation ratio of 58.6% (17/29) for the sites detected by all three methods albeit at a drastic cost in the sensitivity: these sites represented only 1.8% (156/8724) of all sites (Fig. 3d, Additional file 1: Supplementary Tables 7-9). Furthermore, the 194 sites (2.2% of all sites) detected by RED-ML and SPRINT had 36% (9/25) validation ratio, compared to 0% validation ratio (0/8) for the sites detected by SPRINT-only (Fig. 3e, Additional file 1: Supplementary Table 9). Altogether, none of the methods had satisfactory performance in terms of the editing site discovery when considering both the sensitivity and the accuracy even when sites detected by more than one method were tested. Therefore, as described below, we explored additional filtering options based on genomic locations of the candidate sites to preferentially remove false positives (Fig. 1).

### True unannotated editing sites in exonic regions are very rare

**Fig. 4:**
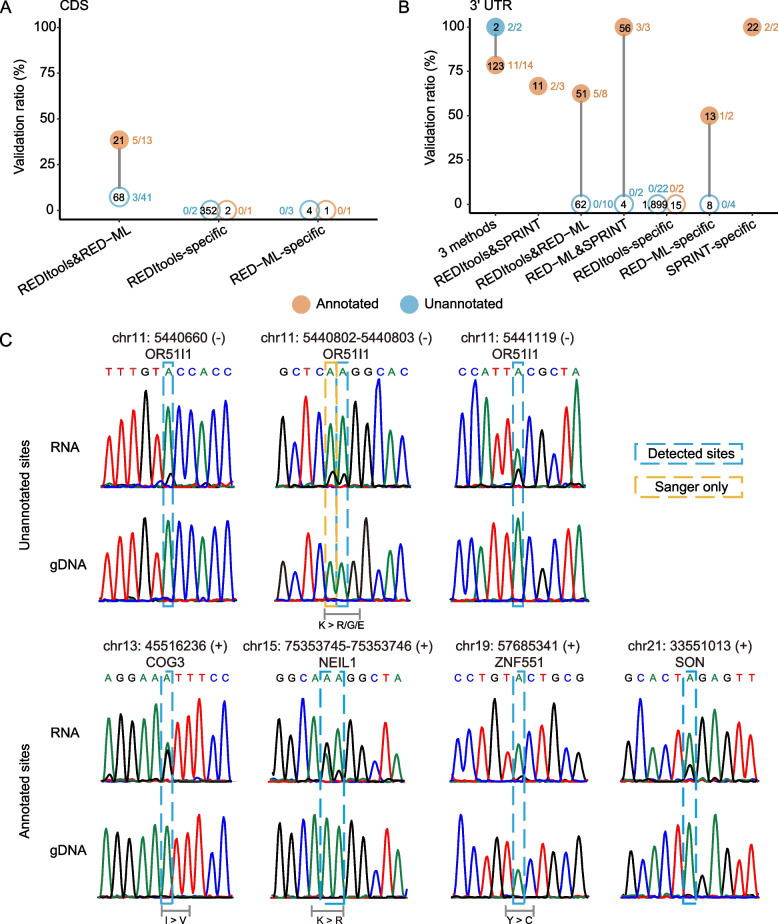
True unannotated ADAR editing events are rare in exonic regions. **a**, **b** Validation ratios of annotated (orange circles) and unannotated (blue circles) candidate edits in **a** CDS or **b** 3′ UTR regions. The number within each circle represents the number of total sites predicted by only one or multiple methods (*X*-axis). The fraction outside each circle represents the corresponding validation ratio. The hollow circles represent sites detected by only one (REDItools-, RED-ML-, or SPRINT-specific) or multiple methods with low (< 7.3%) validation ratios. **c** Sanger sequencing electropherograms of all — 4 unannotated and 5 annotated — validated sites in CDS regions. The sites targeted for validation are demarcated by the blue dashed lines while the nearby unannotated ADAR site found by Sanger only is demarcated by orange dashed lines. Genomic coordinates of all sites and the names of the corresponding genes are shown above the electropherograms with the theoretical amino acid changes caused by the editing shown below. **a**–**c** Only sites with the maximum editing level of > 0.2 across all 130 samples were used in this analysis. Source data are provided as a Source data file

One conspicuous feature of the unannotated RNA editing sites was abundance of sites mapping to exons of annotated genes. Of the 8724 unique unannotated sites, 2735 (31.3%) mapped to the exons, of which 424 mapped to coding regions (CDSs), 1978 mapped to 3′ untranslated regions (UTRs) and 333 to 5′ UTRs. For comparison, only 464 (14.5%) annotated sites mapped to exons of which 32, 291 and 141 mapped to CDSs, 3′ and 5′ UTRs respectively (Additional file 1: Supplementary Tables 7-8 and 10). Considering the importance of editing sites in exonic regions in terms of proteome diversity and gene expression regulation [47, 48], we explored how many of the unannotated sites mapping to these regions were real. Most of the unannotated exonic sites (2529/2735) were found only by REDItools; however, of the 22 tested REDItools-specific sites representing 1899 unannotated sites mapping to 3′ UTRs, none could be validated (Fig. 4b, Additional file 1: Supplementary Table 10). These observations were consistent with the overall low validation ratios of the unannotated sites found by REDItools described above. Furthermore, none of the 10 tested sites representing 62 unannotated sites mapping to 3′ UTRs found by both REDItools and RED-ML could be validated (Fig. 4b, Additional file 1: Supplementary Table 10). Most of the unannotated sites in CDSs were found either only by REDItools (352) or by both REDItools and RED-ML (68) as shown in the Additional file 1: Supplementary Table 10. However, given the failure to validate exonic sites predicted by REDItools only, we focused only on the latter 68 sites since given the above validation ratios, it was very likely that most if not all of the 352 sites were false positives. However, testing 41 sites found by both REDItools and RED-ML returned only 3 positive sites (7.3%, Fig. 4a, Additional file 1: Supplementary Table 10). Interestingly, all 3 sites were located in the coding region of the same gene OR51I1, encoding a member of human olfactory receptor family, which also contained another unannotated site adjacent to one of the 3 validated sites and detected by Sanger sequencing (Fig. 4c). Thus, we could identify 4 unannotated ADAR editing events in the CDS of OR51I1 of which 2 adjacent sites had the potential to change the amino acid from K to R, G or E (Fig. 4c). Altogether, of the total 86 tested unannotated sites in exons, we could confirm only 5 (3 in CDSs and 2 in 3′ UTRs), resulting in the weighted validation ratios of 1.2% and 0.1% in CDS and 3′ UTR regions, respectively.

These results were in a stark contrast with the annotated sites in CDS and 3′ UTR regions where the corresponding validation ratios were much higher (Fig. 4a, b, Additional file 1: Supplementary Tables 7-8 and 10). For example, the validation ratio of the annotated sites detected by both REDItools and RED-ML in CDSs was 38.5% (5/13) compared to only 7.3% (3/41) for the unannotated sites (Fig. 4a, Additional file 1: Supplementary Table 10). The corresponding ratios for the sites in the 3′ UTRs were 62.5% (5/8) vs 0% (0/10, Fig. 4b, Additional file 1: Supplementary Table 10). Finally, while both annotated and unannotated sites detected in exons (specifically, 3′ UTRs) by all three methods had high validation ratios of 78.6% and 100%, they respectively represented 38.1% (123/323) and 0.1% (2/2402) of all annotated and unannotated exonic sites (Additional file 1: Supplementary Table 10). Overall, we estimated the weighted validation ratios in the annotated exonic sites as 33.7% and 75.7% in the CDS and 3′ UTRs. In summary, these results clearly showed that extreme care must be taken when interpreting unannotated human ADAR sites discovered in exons since false positive ratios in those regions can be very high. However, even among the annotated sites, the ones mapping to exons need to be validated, which is especially relevant for the sites in the CDS regions where the validation ratio was much lower than for the rest of annotated sites.

### True unannotated editing sites are common in non-coding transcripts

**Fig. 5:**
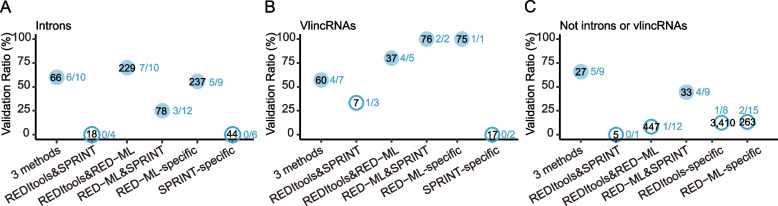
Validation ratios of unannotated ADAR candidate sites in the non-coding regions of the genome. The validation ratios (*Y*-axis) of non-exonic unannotated sites mapping to the same strands of **a** introns of annotated genes, **b** vlincRNAs, but not introns, or **c** elsewhere in the genome are shown. The number within each circle represents the number of total sites predicted by only one (REDItools-, RED-ML-, or SPRINT-specific) or more methods (*X*-axis). The fraction outside each circle represents the corresponding validation ratio. The hollow circles represent sites detected by only one or multiple methods with low validation ratios. **a**–**c** Only sites with the maximum editing level of > 0.2 across all 130 samples were used in this analysis. Source data are provided as a Source data file

As the next step, we explored the authenticity of unannotated ADAR sites in the non-coding genome. We first investigated the 672 sites located in the introns of the annotated genes where a majority of non-coding transcripts, both by relative mass and sequences complexity, were previously found [49]. Overall, we achieved good validation ratios for most methods, as high as 70% (7/10) for the 229 sites detected by both REDItools and RED−ML (Fig. 5a, Additional file 1: Supplementary Tables 7-8 and 11). The weighted validation ratio of all intronic unannotated sites was 52.2% based on testing 51 sites. We then explored the 272 sites, which excluded the sites predicted by REDItools only, found in the very long intergenic non-coding (vlinc) RNAs, a widespread class of long non-coding (lnc) RNAs that covers on the order of 10% of the human genome [50] and accounts for as much as half of non-polyadenylated RNA in the nucleus [51]. These transcripts were implicated in control of gene expression in *cis* and *trans* [45], cellular senescence [52], and share common features with ASAR genes encoding lncRNAs that control replication timing of human chromosomes [53]. After testing 20 unannotated vlincRNA sites, we could achieve even higher overall weighted validation ratio of 79.9% (Fig. 5b, Additional file 1: Supplementary Table 11).

The remaining 48.1% (4194/8724) of the unannotated sites mapping to the non-coding genome were mostly detected by either only REDItools (3410) or RED-ML (263) or by both methods (447, Additional file 1: Supplementary Table 11). However, the corresponding validation ratios were very low: 12.5% (1/8), 13.3% (2/15), and 8.3% (1/12, Additional file 1: Supplementary Table 11). The only high validation ratios were observed for the small groups of 27 and 33 sites detected either by all three methods or by both RED-ML and SPRINT — respectively 55.6% (5/9) and 44.4% (4/9, Fig. 5c, Additional file 1: Supplementary Table 11). The overall weighted validation ratio of the unannotated sites outside of introns and vlincRNAs was only 12.6%.

### Genomic landscape of A-to-I editing sites in K562

**Fig. 6:**
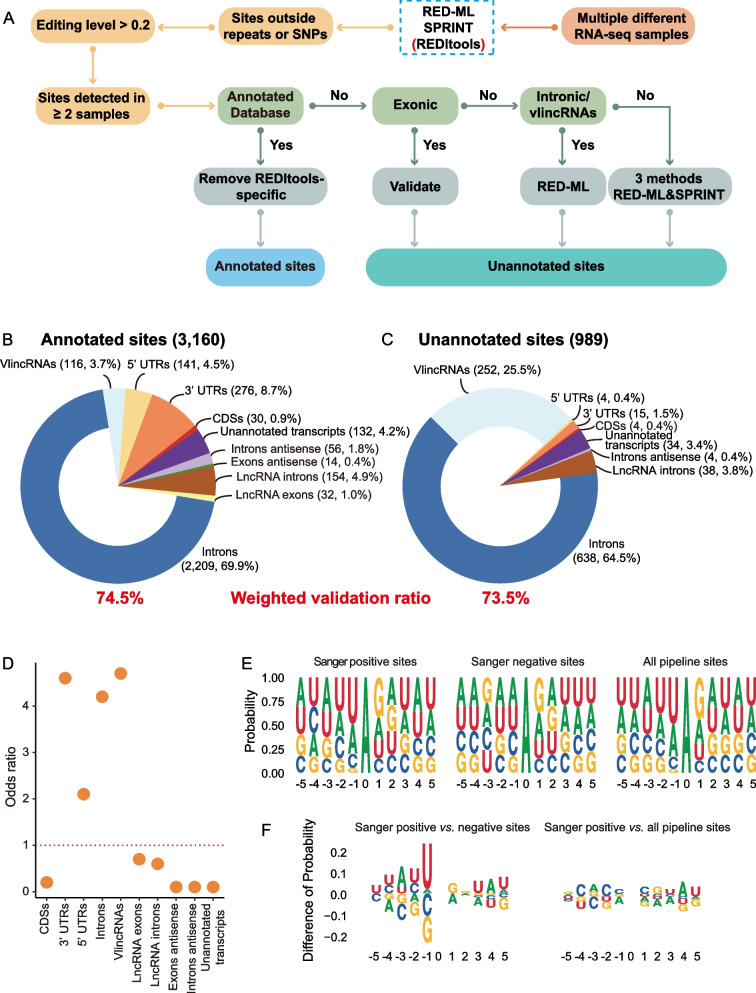
Genomic landscape of ADAR editing events in a cancer cell line. **a** Flow chart diagram illustrating the process of obtaining true annotated and unannotated editing sites in the K562 cancer cell line. As shown in this study, sites detected only by REDItools and not supported by other methods have to be interpreted with caution due to very low validation ratios. **b**, **c** Pie charts showing distributions of true annotated (**b**) and unannotated (**c**) editing sites across the indicated genomic elements. The two numbers for each elements represent the number of editing mapping within each element and the fraction of the total sites. **d** Odds ratios of enrichment of the true annotated K562 sites in various genomic elements. The red dashed line represents the odds ratio of 1. **e** Sequence motifs around the sites positive or negative in the Sanger validation and all final sites predicted by the pipeline shown in the **a**–**c**. The editing site is represented by the position “0.” **f** Differences in the fraction of each base at each position. The positive values on the *Y*-axes mean enrichment in the sites positive in the Sanger validation. **b**–**d** Only sites with the maximum editing level of > 0.2 across all 130 samples were used in this analysis. Source data are provided as a Source data file

The results above suggested that after filtering un-annotated sites by their genomic locations and by the method of detection, it is possible to generate a list of true unannotated sites with high validation ratio. Figure 6a illustrates the pipeline developed based on the results above to obtain the lists of true annotated and unannotated editing sites and, thus, the overall compendium of authentic ADAR editome of the K562 cancer cell line. As shown above, annotated and unannotated sites predicted only by REDItools without support from other methods had very low validation ratios and had to be excluded. Thus, we excluded 36 annotated sites detected only by REDItools that had validation ratio of 0% (0/8, Fig. 3d, e, Additional file 1: Supplementary Tables 7-9) from the 3196 sites to obtain 3160 annotated sites that represented a union of sites detected by RED-ML and SPRINT and had weighted validation ratio 74.5% (Fig. 6b). The unannotated sites required additional filtering steps based on the genomic location and methods: sites mapping to introns or vlincRNAs could be reliably called using RED-ML only; however, sites mapping elsewhere had to be also predicted by SPRINT (Fig. 6a). Using this approach, we could obtain 932 unannotated sites. In addition, during the process of Sanger validation, we discovered additional 57 unannotated sites adjacent to the sites being validated as illustrated in Fig. 3b. Thus, in total, we discovered 989 unannotated sites with the weighted validation ratio of 73.5% (Fig. 6c). Overall, we could detect 4149 high quality K562 ADAR editing sites with the overall validation ratio of 74%. Since selection of the unannotated sites was biased by the genomic regions, we analyzed the properties of annotated and unannotated editomes separately.

Analysis of both editomes showed that ADAR editing sites that have the potential to increase the proteome diversity are very rare in human cancer cells. Totally, we predicted 34 high confidence editing sites that map to CDSs in K562 cells in our editome compendium (Fig. 6b, c), of which we could directly validate by Sanger only 9 — 5 annotated and 4 unannotated sites — in 5 genes, of which 6 can cause amino acid changes (Fig. 4c). Ten out of the 34 sites in CDS regions failed Sanger validation (Additional file 1: Supplementary Tables 7-8 and 10); therefore, the true number of editing events in the coding regions could be even lower. Based on the annotated sites, we estimate that the sites in CDSs represent no more than 1% of all non-repeat editing sites in K562 (Fig. 6b). On the other hand, sites that map to 3′ UTRs are much more common, and we estimate that they represent up to ~10% of the total non-repeat editome of the human cancer cells (Fig. 6b). Furthermore, based on the annotated editome, editing sites were enriched in 3′ UTRs with the odds ratios of 4.6 (Fig. 6d, Additional file 1: Supplementary Table 12). On the other hand, the enrichment in the 5′ UTR was not that high with the odds ratio of 2.1 (Fig. 6d, Additional file 1: Supplementary Table 12).

The majority of annotated (85.9%) and unannotated (97.7%) ADAR editing sites mapped to the non-coding transcripts, the so-called RNA 'dark matter' [46, 54–56]. Sites mapping to the intronic regions represented the majority (~2/3) of both annotated and unannotated sites amounting to 2847 total sites (68.6%, Fig. 6b, c, Additional file 1: Supplementary Table 13). The annotated ADAR editing events were found in introns of 799 genes with additional 332 genes containing unannotated sites discovered in this work. Interestingly, vlincRNAs represented relatively untapped reservoir of novel sites with 25.5% (252/989) of unannotated sites mapping to those transcripts compared to only 3.7% (116/3160) annotated sites (Fig. 6a, b, Additional file 1: Supplementary Table 13). Overall, 368 (8.9%) of all sites were found in 87 vlincRNAs (Fig. 6b, c, Additional file 1: Supplementary Table 13). In general, we found strong preference for the true editing sites to occur in the introns and vlincRNAs as evidenced by the respective odds ratios of 4.7 and 4.2 based on the annotated sites (Fig. 6d, Additional file 1: Supplementary Table 12, the unannotated sites were excluded from this calculation due to the selection bias).

Of the remaining sites, 32 and 192 respectively mapped to exons and introns of annotated lncRNAs, of which 38 sites mapping to the latter were unannotated (Fig. 6b, c, Additional file 1: Supplementary Table 13). The remaining 240 sites, including 34 unannotated sites, mapped to totally unknown transcripts (Fig. 6b, c, Additional file 1: Supplementary Table 13). Interestingly, among those, 14 and 60 sites corresponded to novel transcripts that were antisense to respectively exons and introns of annotated genes (Fig. 6b, c, Additional file 1: Supplementary Table 13). However, editing sites were significantly enriched only in introns, vlincRNAs, 3′ UTRs, and, to a lesser extent, in 5′ UTRs, but not in the other non-coding regions (Fig. 6d, Additional file 1: Supplementary Table 12). Furthermore, while intronic transcripts and, especially vlincRNAs, appear to harbor unannotated sites, our discovery efforts have shown that most of ADAR sites in 3′ or 5′ UTRs might have already been annotated. All these 3160 annotated sites and 989 unannotated sites were listed in Additional file 1: Supplementary Tables 14 and 15.

Human ADARs have been shown to have preference for uracil (U) in the -1 position (the base immediately 5′ to the editing site) [57]. Therefore, we tested whether we could find this and/or any other differences in the motifs immediately flanking positive and negative editing sites found in this work. Strikingly, we found a clear preference for U in the − 1 position in the 143 sites that were positive in the Sanger validation compared to the 357 sites where no editing was detected by Sanger (Fig. 6e, f, Additional file 1: Supplementary Table 16). Furthermore, we found pronounced enrichment of U in the − 1 position of all 4149 (3160 + 989) sites predicted by our pipeline just like for the Sanger positive sites, further supporting the reliability of the final list of predicted sites.

### High fractions of potentially false positive ADAR sites in the public databases

**Fig. 7:**
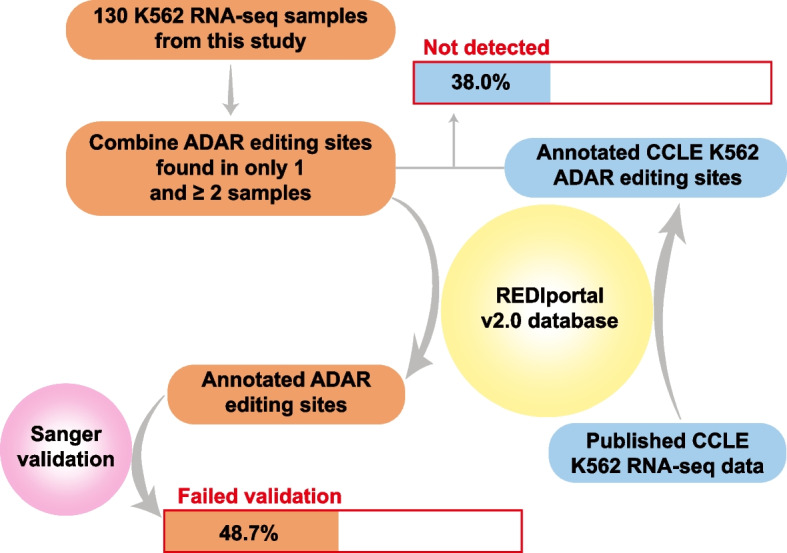
Estimates of the minimal fractions of false positive non-repeat ADAR editing sites in public databases. The estimates were provided based on two approaches: (1) failed Sanger validation of annotated ADAR sites detected in at least 1 sample and (2) annotated sites detected in published K562 CCLE (The Cancer Cell Line Encyclopedia) RNA-seq data that could not be reproduced in any of the 130 K562 RNA-seq samples used in this study. Source data are provided as a Source data file

As mentioned above, the validation ratios for the various types of annotated editing sites were consistently higher than those for the unannotated ones. Still, out of the 3160 annotated editing sites detected in this work, we failed to validate 25.5%, leaving the question of the authenticity of these ~806 sites open. Furthermore, it is important to stress that the 3160 annotated sites were independently detected by different investigators in different cell types and by us in at least two different K562 samples. As such, it is almost certain that there is an additional hidden population of false annotated sites that were not detected in this work and therefore not even subjected to Sanger validation. Therefore, to account for such sites, at least partially, and to estimate the minimal number of potential false positives, we included 2630 annotated sites detected in just one sample out of which, based on the results of validation, we expect 2012 or 76.5%, to be false (Fig. 7). Taken together, we estimated the total fraction of annotated sites in K562 that would fail validation to be at least 48.7% ((806 + 2012)/(3160 + 2630)) (Fig. 7).

Such potential false positive sites could represent hotspots of errors caused by, for example, PCR amplification, cDNA synthesis, or NGS. Alternatively, they could represent real ADAR sites in some samples other than K562 while still represent hotspots of errors in K562. To account for this possibility, we limited the analysis to the sites that are currently deposited in the public databases and found in K562. For this purpose, we used a K562 RNA-seq dataset from the Cancer Cell Line Encyclopedia (CCLE), which is the same dataset that was used to derive annotated K562 sites in the CLAIRE (Cell Line A-to-I RNA Editing) database which is a part of REDIportal [23] (Fig. 7). We performed the editing site prediction in the CCLE K562 sample using the same analytical procedure as in the CLAIRE publication [23] and found 244 sites with the editing level > 0.2, most of which (237/244 or 97.1%) were also annotated in the REDIportal v2.0 database [22]. Of those, a large proportion (38.0%, 90/237) could not be detected in any of our 130 K562 samples by any of the three methods (Fig. 7, Additional file 1: Supplementary Table 17). It is important to emphasize that the 38.0% refers to the annotated K562 sites that failed to be reproduced at the level of site calling, even prior to Sanger validation, and thus this fraction represents the minimal estimate of questionable editing sites which is also consistent with the higher minimal estimate of 48.7% based on Sanger validation. Overall, these results strongly argue that a large fraction of questionable ADAR sites does exist in the public databases.

These surprising results could be explained by the high false positive rates for the newly discovered sites found by the REDItools methods used to generate millions of annotated sites [23, 24]. Therefore, we also tested a new version of this program, HPC-REDItools [58], using the same pipeline as for the other 3 methods. As shown in the Additional file 6: Supplementary Figure 5a, b, HPC-REDItools detected the largest number of sites unique to this method — respectively 4715/10,484 (45.0%) and 42,513/63,817 (66.6%) annotated and unannotated sites. To determine the accuracy of these predictions, we tested 21 and 17 annotated and unannotated HPC-REDItools-specific sites by Sanger and could confirm respectively 11 (52.4%) and 2 (11.8%) as shown in the Additional file 3: Supplementary Figure 2 and Additional file 6: Supplementary Figure 5c (also see Additional file 4: Supplementary Figure 3, Additional file 5: Supplementary Figure 4, Additional file 1: Supplementary Tables 7-8).

Even though HPC-REDItools was more sensitive in the detection of the annotated sites than the other 3 methods, the validation rate for those sites was lower than those for sites found only by RED-ML (81%) or SPRINT (71.4%) (Fig. 3e, Additional file 1: Supplementary Table 9) and for the annotated sites detected by our pipeline (74.5%). HPC-REDItools performed better than REDItools in terms of the specificity of detecting unannotated sites unique to each method with the corresponding validation ratios 11.8% and 3.1% (Additional file 6: Supplementary Figure 5c, Additional file 1: Supplementary Tables 7-8). However, the validation ratio was still very low for the new version, especially considering the large number of high false positive unannotated sites generated by HPC-REDItools, suggesting that the unannotated sites obtained by this method need to be further refined. Therefore, in this study, the sites predicted only by HPC-REDItools were not used for downstream analyses.

The failure to validate so many annotated and unannotated sites prompted us to further evaluate the sensitivity of Sanger validation which could be influenced by the abundance of the corresponding transcripts and by the editing levels of the tested sites. As mentioned above, the former consideration should not influence the validation rates found in this work since only sites for which we could obtain clear RT-PCR products and Sanger electropherograms were considered in this work. However, we have further explored the effects of transcript abundance and editing levels on the validation rates. We estimated the editing level and transcript abundance of each of the 500 sites tested by Sanger in the actual sample subjected to validation. The transcript abundance was estimated based on the coverage of total RNA-seq reads (representing edited and not edited transcripts) mapping to the corresponding site.

As can be seen in the Additional file 7: Supplementary Figure 6, the median abundances and editing levels for the two groups of sites were close: the median read depths for respectively the positive and negative sites were 21 and 15, while the median editing levels were 0.53 and 0.50. We then applied different thresholds of read depths and editing levels and found that increasing the read depth or editing levels of sites had no effect on the fraction of sites positive in Sanger (Additional file 7: Supplementary Figure 6). Therefore, these features unlikely affected the conclusions from the validation tests.

### Evaluating predictions of ADAR sites using genomic sequences

The availability of multiple true positive and negative ADAR sites from the Sanger validation has also allowed us to test feasibility of predicting such sites solely based on genomic sequences, bypassing the need to generate costly RNA-seq data. Recently, a Deep Learning-based method, EditPredict, that used Convolutional Neural Network models has been developed for such purpose [59]. However, the application of EditPredict on the sites obtained from this work has shown that while promising, this method is still far from being a practical tool for prediction of ADAR sites. Of the unannotated sites that were positive and negative in the Sanger validation, EditPredict (the webserver version) predicted that 19/55 (34.5%) and 68/280 (24.3%) represented ADAR sites, respectively. The corresponding numbers were 40/88 (45.5%) and 34/77 (44.2%) for the annotated sites. The higher fractions for the annotated sites and the inability to differentiate between the true and false annotated ADAR sites were most likely due to the fact that EditPredict was trained on the publicly available annotated sites. Therefore, only the results obtained for the unannotated sites represent fair estimate of the performance of this method.

Overall, the fraction of the sites predicted by EditPredict was higher for the true ADAR sites validated by Sanger (34.5% vs 24.3%). However, these results also demonstrated the problems with the method. First, the sensitivity of detection was relatively low (< 50%) even for the annotated sites. Second, and more importantly, the specificity of the method was also low, which would result in generation of a significant fraction of false positive sites. These results illustrated that, while promising, this and similar methods require further development and cannot yet substitute for methods based on analysis of the wetlab data such as RNA-seq. The reasons for this likely include (1) binding of ADARs to their substrates strongly depends on proper RNA folds that are still challenging to model [43], and (2) in addition to substrate recognition by ADARs, editing also depends on interaction with other factors as mentioned above.

## Discussion

In this work, we focused on generating a compendium of authentic ADAR sites in a human cell line K562 with the goals of (1) illustrating technological challenges that could arise during such an endeavor due to the current state of sequencing and analytical techniques; (2) providing potential solutions to these problems; and (3) exploring possible biological implications of analyzing cancer editome. The unique aspect of our work is extensive independent validation of the results of the analytical editing site prediction pipeline using the highly accurate Sanger sequencing of 500 of predicted editing sites. Such magnitude of independent validation is very rare in genome-wide ADAR prediction studies but, as the results presented here have shown, is essential since the fraction of false positives can be very high. Surprisingly, even already annotated sites appear to contain a large fraction, > 40%, of questionable editing events that cannot be validated even in the same cell type. This fraction is much higher among the newly discovered sites and can reach over 90% depending on the analytical method used. It is, however, theoretically possible that some of the annotated K562 sites that failed to be detected in this work do in fact represent real ADAR sites that are edited only under very specific biological conditions. Still, at the very least, our results suggest that interpreting results obtained from genome-wide editome surveys has to be done with caution even when using sites deposited in the public databases.

It is theoretically possible that the failure to validate many candidate editing sites could be due to a low sensitivity of our validation method. However, we think that this is unlikely for the following reasons. First, we only considered sites for which we could obtain high-quality Sanger electropherograms with low background as illustrated in the Figs. 3b and 4c. Therefore, sites for which the validation procedure has not worked or has not yielded specific products were not considered as tested and have not entered into the calculations of the validation ratios. Still, it is conceivable that some true sites failed to be validated due to preferential loss of edited transcripts during reverse transcription, PCR, or some other steps. However, in this respect, it is important to emphasize that we used the same validation conditions for all different types of sites — those detected in only one or multiple samples, found by different analytical methods and so on — and obtained validation ratios that ranged from 5% to over 70%. It is hard to imagine that the sensitivity would vary significantly among the different groups of sites and, therefore, it is much more likely that, indeed, different types of sites contain very different fractions of true positives.

Also, it is possible that true ADAR sites that occur only in specific subpopulations of cells would result in very low overall editing levels in the bulk cell population that would be below the level of detection by the Sanger validation. However, in this work, we limited our survey to editing sites, both annotated and unannotated, that had editing levels > 0.2 which should be detectable on the Sanger sequencing traces [37]. Taken together, these observations suggest that the current analytical methods developed to predict ADAR sites from RNA-seq data are still far from perfect, even though one of them, RED-ML, consistently performed better than the others (see below). Strikingly, REDItools and HPC-REDItools which were used for discovery of millions of annotated sites in the public databases [22–24], had the largest numbers of sites with very low overall accuracies, potentially explaining the existence of so many questionable sites in these databases.

To address this issue, in this study we have identified potential solutions that adopted in an analytical pipeline shown in the Fig. 6a that could increase the fraction of true positive sites, even though each such option also comes at a price. First, detection of sites in multiple independent samples of the same cell type can significantly increase the authenticity of editing sites. However, reliance on multiple samples would increase the cost of the experiment and prohibit editing site detection in situations where multiple independent samples for the same cell type or biological state are not available. Second, as shown in the Fig. 6a, we recommend a pipeline in which unannotated candidate sites are filtered based their genomic locations in combination with the analytical method(s) by which they were detected to maximize the authenticity ratio. However, such filtration would limit the genomic space of the discovered sites to mostly introns and vlincRNAs. Third, at least in a human cancer system, it appears that true unannotated exonic sites are rare and therefore all such candidate sites derived from RNA-seq data should be ideally subjected to Sanger validation before their inclusion into the overall analysis, which would of course increase the cost and time of the experiment. Therefore, while these considerations provide practical guidelines for editome profiling and discovery, we believe that they also make a very strong case for additional development of improved analytical techniques that can authentically detect editing sites.

## Conclusions

One of the most striking conclusions from this study is that ADAR sites that can recode mRNAs to make novel proteins are very rare in human cells, consistent with at least one previous study [60]. Moreover, most of unannotated candidate sites mapping to coding regions, as well as other portions of mature mRNAs, are likely to be false positives. This could potentially call into question some previous conclusions that concern effects of cancer editomes on the proteome diversity of human cancer cells. Still, our results show that while sparse, novel recoding editing sites do exist and thus warrant additional discovery efforts due to their potential importance, providing additional arguments for development of improved analytical tools for accurate and sensitive editing site discovery.

On the other hand, it is fairly clear from this work, that most of the newly discovered true human editing sites would likely occur in non-coding transcripts, the RNA 'dark matter' [46, 54–56]. Since, for most part, function and biological relevance of such transcripts are not known [51, 61, 62], the biological importance of most editing sites in these transcripts is also unclear. This raises a natural question of whether discovery of editing sites in such transcripts is even justified as opposed to shifting focus only to sites that can either recode proteome or at the very least occur in non-coding portions of mature mRNAs since biological relevance of such sites is easier to rationalize. We believe, however, that ongoing discovery of sites in the RNA 'dark matter' is important for at least two reasons. First, compendium of real, validated ADAR editing sites can be extremely valuable for training new and improved algorithms, potentially relying on deep learning approaches that have shown great potential in the highly complex problems of RNA and protein structure prediction [63–66]. In this respect, interestingly, the method based on machine learning RED-ML [27] performed better than the other 2 techniques, suggesting that deep-learning-based methods could indeed represent the future editing site discovery, based on both analysis of the RNA-seq data and for prediction of editing sites based solely on the genomic sequence. Second, disease-associated editing sites, even those whose function we do not understand, can represent a source of potential biomarkers [67, 68].

## Methods

### Source of the RNA-seq data

The details of RNA-seq samples used in this study are listed in the Additional file 1: Supplementary Table 1 together with the corresponding GEO accession numbers. The human CML cell line K562 used in this work was obtained from Cell Bank of Chinese Academy of Sciences. The cell line and its derivatives were maintained in RPMI 1640 medium (ExCell Bio) supplemented with 10% (v/v) fetal bovine serum (Thermo Fisher Scientific, US) and 1% (v/v) pen/strep (Thermo Fisher Scientific, US) at 37 °C in 5% CO_2_. For drug treatments, K562 cells (5 × 10^5^ cells/ml) were cultured in 6-well plates using 3 ml of the medium per well. After 16 h, drugs or DMSO/water controls were added at the different concentrations and for various amounts of time as listed in the Additional file 1: Supplementary Tables 1 and 2. Total RNA was isolated with TRNzol (TIANGEN, Beijing) and used to construct of RNA-seq libraries after removing rRNAs with Ribo-Zero™ kit. The RNA-seq library preparation and sequencing was outsourced to Novogene corporation (Beijing). Sequencing was performed using the Illumina HiSeq X Ten platform and paired-end 150-bp (PE150) strategy on a 10-gigabase (GB) scale.

### Genome resequencing of K562 cell line

Genomic DNA from our K562 clone was resequenced on the Illumina NovaSeq platform using the PE150 strategy and 90-GB scale by Novogene Corporation (Beijing). Only reads that passed quality filtering were aligned to the human genome by BWA (version: 0.7.8-r455) and then further filtered for by Samblaster (version 0.1.21) to remove improper alignments. Duplicate reads were removed by SAMtools (version 1.0) and Sambamba (version 0.4.7). Finally, the remaining alignments were used for variant calling by SAMtools. The above analysis was done by Novogene Corporation (Beijing).

### RNA-editing sites detection and validation

Quality filtered NGS reads were trimmed with fastx toolkit (version 0.0.13) [69] and aligned to the human genome (GRCh38/hg38) by Tophat2 (version 2.1.1) [70, 71]. PCR duplicates were removed using Picard suite (version 2.0.1). The alignments were sorted and indexed by SAMtools (version 1.9) [72, 73] and used for candidate RNA editing sites detection by REDItools [25], RED-ML [27], and SPRINT [26]. Each RNA-seq sample was processed independently by each of the three analytical methods. REDItools [25] was run with the parameters “-m 50 -u -T 6-0 -n 0.0.” RED-ML [27] and SPRINT [26] were run using the default parameters. In addition, the new version of REDItools, HPC-REDItools [58], was also employed using the following parameters: -q 40 -bq 30 -mbp 6 -Mbp 6.

Putative ADAR editing sites were filtered against the common SNPs from dbSNP 151 [74] and K562 in-house SNPs to remove sequence variants. The sites mapping to repeats as annotated by the RepeatMasker track [75] of the UCSC Genome Browser were removed. The remaining sites were then filtered based on their mappability scores as defined by the “Mappability or Uniqueness of Reference Genome from ENCODE” track [76, 77] from the UCSC Genome Browser [78]. Only the sites with a mappability score of 1 based on 100mer alignability and an average mappability score of > 0.5 calculate based on the 24mer alignability in the ± 150 bp window around the sites were kept.

The editing level for each site in each sample was determined by each method. If a site was detected by multiple methods in the same sample, its editing level was calculated as the average of editing levels estimated by each method that detected it. RNA editing candidates with the maximum editing level among the 130 samples of > 0.2 and found in at least two samples by any method were used for the downstream analyses. A site was considered to be found by only one method if it was detected in all samples using that method only. If a site was found by more than one method in different samples, it was considered as found by different methods even if the editing in the sample where the editing level passed the threshold was found only by one method. In other words, if a site was found in one sample using RED-ML with editing level of 0.1 and in another sample using SPRINT with the editing level of 0.3, it was considered as detected by both of these methods even though only one method detected it with the editing level exceeding the threshold of 0.2.

Specific editing sites were validated by PCR amplifying 146–593 bp regions containing the sites from the corresponding RNA samples and the K562 genomic DNA using nested primers. The PCR products were purified by VAHTS DNA clean beads (Vazyme, Nanjing) and then sequenced directly using Sanger platform by Sangon Biotech (Shanghai) and Biosune (Xiamen). The resulting electropherograms were scored manually for the presence of true editing sites. Only electropherograms with low background were considered in the analysis.

### Overlap with the annotated editing sites and various genomic features

The annotated ADAR RNA-editing sites were downloaded from the following databases: (1) REDIportal v2.0 [22], (2) RADAR [20], and (3) DARNED [21]. Known genes and lncRNAs were downloaded from GENCODE release 41(GRCh38.p13)) [79]. The coordinates of 407 vlincRNAs identified in K562 cell line were taken from St. Laurent et al. [50].

ADAR sites located within exons or introns of known genes or lncRNAs and vlincRNAs had to map to the same genomic strands as the corresponding transcripts. When necessary, the coordinates of datasets were converted from HG19 to HG38 using the LiftOver tool from the UCSC Genome Browser. The overlaps between the RNA-editing sites and the different genomic element were calculated using the “intersect” function of the BEDTools (version 2.30.0) [80, 81].

### The motif analysis

The sequence within ± 5 bp region around each site was extracted using the “getfasta” function of BEDTools [80]. Then, the fraction of each base (A, C, G, U) at each coordinate in the ± 5 bp region was calculated. Finally, the plot was generated by the R package “Logoplot.”

## Supplementary Information

**Additional file 1:** **TableS1.** Description of the 130 RNA-seq samples. **Table S2.** Description of the drugs usedto treat cells in the RNA-seq analyses. **TableS3.** Fractions of candidate sites located in repeats/Alus. **Table S4.** The numbers of candidatesites predicted by different methods. **TableS5.** The number of candidate sites predicted in only one or at least twosamples with the maximum editing level >0.2. **Table S6.** The validation ratios of candidate sites detected in only1 or at least 2 samples. **Table S7.**The list of sites that were positive in the Sanger validation. **Table S8.** The list of sites that werenegative in the Sanger validation. **TableS9.** The validation ratios of candidate sites detected by one or moremethods. **Table S10.** The validationratios of candidate sites in exonic regions. **Table S11.** The validation ratios of unannotated candidate sites innon-exonic regions. **Table S12.** Theodds ratios of enrichment of the 3160 annotated editing candidate sites indifferent genomic features. **Table S13.**Genomic landscape of RNA editing candidate sites with the maximum editing level>0.2 and detected in at least 2 samples. **Table S14.** List of the 3160 annotated sites. **Table S15.** List of the 989 unannotated sites. **Table S16.** Sequence motifs around the sites positive or negative inthe Sanger validation and all sites predicted by the pipeline. **Table S17.** Overlap between the 237annotated editing sites predicted in the CCLE K562 RNA-seq samples with thesites predicted in our 130 K562 RNA-seq samples.

**Additional file 2:** **SupplementaryFigure 1.** Schematics of the initial ADAR site identificationpipeline used in this study. The pipeline shown here was used to generateannotated and unannotated sites shown in the Figure 2 prior to the subsequentrefinement based on the Sanger validation. The diagram shows the 3 major stepsin pipeline: (1) the initial calling of the candidate editing sites fromRNA-seq data, (2) filtration of false positives and (3) overlap with annotateddatabases.

**Additional file 3:** **SupplementaryFigure 2.** Distribution of sites subjected to the Sangervalidation. Source data are provided as a Source data file.

**Additional file 4:** **SupplementaryFigure 3.** Sanger electropherograms of sites that were positivein the Sanger validation. Electropherograms for the target sites (blue dashedboxes) detected by each method only or by multiple methods are shown for PCRproducts amplified from RNA or genomic DNA (gDNA). Adjacent editing sitesidentified by Sanger only are shown in yellow dashed boxes.

**Additional file 5:** **SupplementaryFigure 4.** Sanger electropherograms of sites that were negativein the Sanger validation. Electropherograms for the target sites (blue dashedboxes) detected by each method or by multiple methods are shown for PCR productsamplified from either (1) only RNA for the sites that did not show evidence ofRNA editing, or (2) both RNA and genomic DNA (gDNA) for the sites that turnedout to represent sequence variants. Adjacent editing sites identified by Sangeronly are shown in yellow dashed boxes.

**Additional file 6:** **SupplementaryFigure 5.** Comparison of HPC-REDItools and the 3 methods(REDItools, RED-ML, SPRINT) used in our pipeline. Venn diagrams representingthe number of annotated (a) and unannotated (b) sites which were detected byone or more different methods in at least 2 samples. (c) Validation ratios(Y-axis) of the annotated (left) and unannotated (right) sites detected byREDItools only (grey), HPC-REDItools only (dark blue) and the pipeline (red).The values in the bars represent the total number of corresponding sites.Source data are provided as a Source data file.

**Additional file 7:** **SupplementaryFigure 6.** Distribution of transcript abundance and editinglevel of all sites subjected to Sanger validation. (a) Read depths in the lnscale and (b) editing levels of sites that were positive (left) and negative(right) in the Sanger validation. The red dashed lines represent the numbers ofreads or editing levels for each quartile. The percentages on the right representthe validation ratios of sites which have read depths or editing levels thatare higher than those represented by the red dashed lines. Source data areprovided as a Source data file.

## Data Availability

All data generated or analyzed during this study are included in this published article, its supplementary information files, and publicly available repositories. The NGS data were submitted to GEO with accession number GSE222170 [82]. Source data of all figures were provided as a Source data file. Custom scripts for the pipeline used to generate RNA editing sites from the fastq files are available from GitHub and Zenodo [83, 84].

## Abbreviations

A: Adenosine
ADAR: Adenosine deaminases acting on RNA
CCLE: Cancer Cell Line Encyclopedia
CDS: Coding DNA sequence
dsRNA: Double-stranded RNA
G: Guanosine
I: Inosine
lncRNA: Long non-coding RNA
NGS: Next-generation sequencing
CML: Chronic myeloid leukemia
U: Uracil
UTR: Untranslated region
vlincRNAs: Very long intergenic non-coding RNA
