# Supplementary material for: Lessons from discovery of true ADAR RNA editing sites in a human cell line
Source: BMC Biol. 2023 Jul 19;21:160. doi: 10.1186/s12915-023-01651-w (PMC10357658; doi:10.1186/s12915-023-01651-w)
Supplement: Supplementary file 2 — Additional file 2: SupplementaryFigure 1. Schematics of the initial ADAR site identificationpipeline used in this study. The pipeline shown here was used to generateannotated and unannotated sites shown in the Figure 2 prior to the subsequentrefinement based on the Sanger validation. The diagram shows the 3 major stepsin pipeline: (1) the initial calling of the candidate editing sites fromRNA-seq data, (2) filtration of false positives and (3) overlap with annotateddatabases. [file 12915_2023_1651_MOESM2_ESM.pdf]

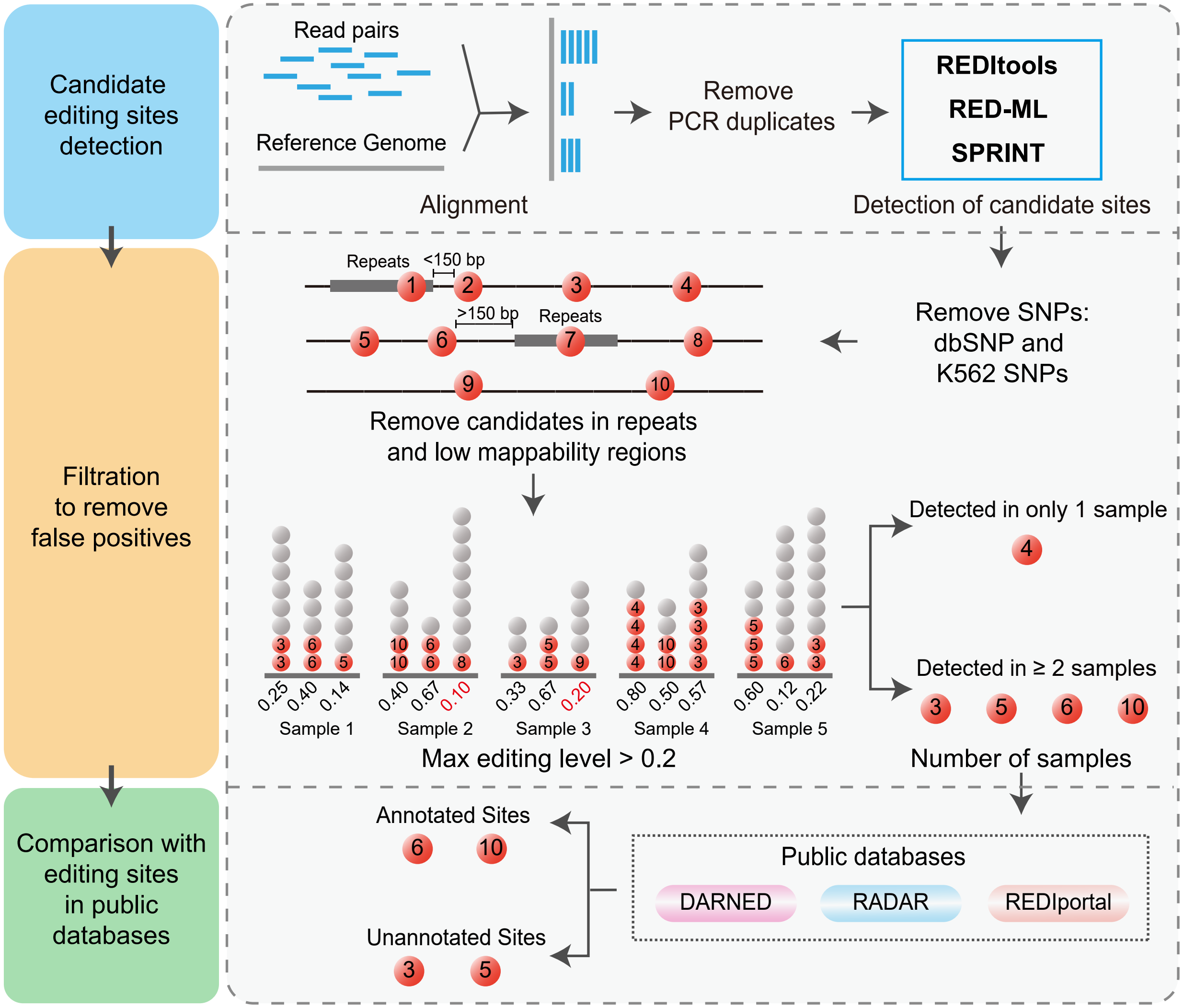

Additional file 2: Supplementary Figure 1. Schematics of the initial ADAR site identification pipeline used in this study. The pipeline shown here was used to generate annotated and unannotated sites shown in the Figure 2 prior to the subsequent refinement based on the Sanger validation. The diagram shows the 3 major steps in pipeline: (1) the initial calling of the candidate editing sites from RNA-seq data, (2) filtration of false positives and (3) overlap with annotated databases.
