# Supplementary material for: Lessons from discovery of true ADAR RNA editing sites in a human cell line
Source: BMC Biol. 2023 Jul 19;21:160. doi: 10.1186/s12915-023-01651-w (PMC10357658; doi:10.1186/s12915-023-01651-w)
Supplement: Supplementary file 3 — Additional file 3: SupplementaryFigure 2. Distribution of sites subjected to the Sangervalidation. Source data are provided as a Source data file. [file 12915_2023_1651_MOESM3_ESM.pdf]

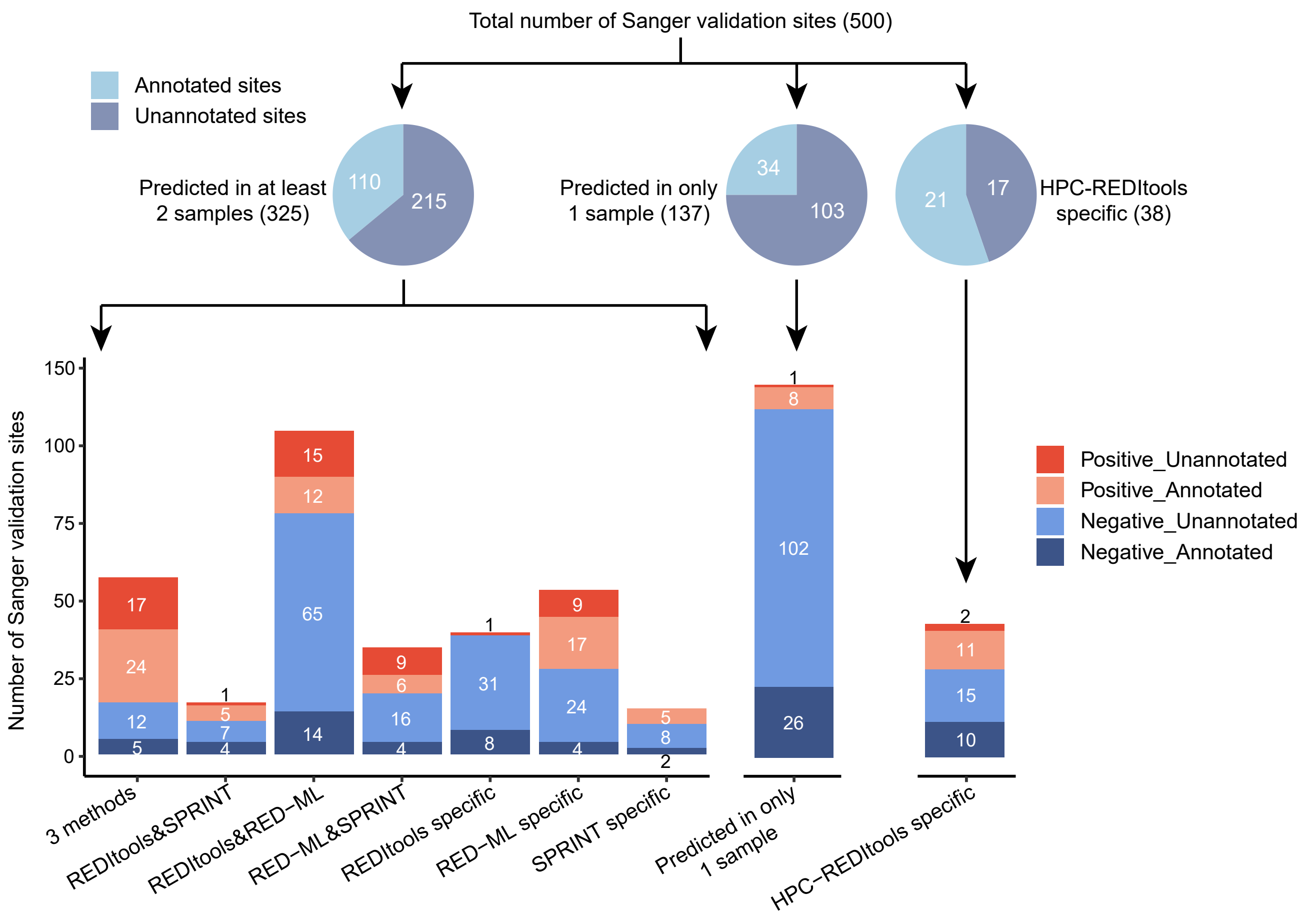

Additional file 3: Supplementary Figure 2. Distribution of sites subjected to the Sanger validation. Source data are provided as a Source data file.
