# Supplementary material for: Lessons from discovery of true ADAR RNA editing sites in a human cell line
Source: BMC Biol. 2023 Jul 19;21:160. doi: 10.1186/s12915-023-01651-w (PMC10357658; doi:10.1186/s12915-023-01651-w)
Supplement: Supplementary file 4 — Additional file 4: SupplementaryFigure 3. Sanger electropherograms of sites that were positivein the Sanger validation. Electropherograms for the target sites (blue dashedboxes) detected by each method only or by multiple methods are shown for PCRproducts amplified from RNA or genomic DNA (gDNA). Adjacent editing sitesidentified by Sanger only are shown in yellow dashed boxes. [file 12915_2023_1651_MOESM4_ESM.pdf]

Additional file 4: Supplementary Figure 3. Sanger electropherograms of sites that were positive in the Sanger validation. Electropherograms for the target sites (blue dashed boxes) detected by each method only or by multiple methods are shown for PCR products amplified from RNA or genomic DNA (gDNA). Adjacent editing sites identified by Sanger only are shown in yellow dashed boxes.

3 methods - annotated

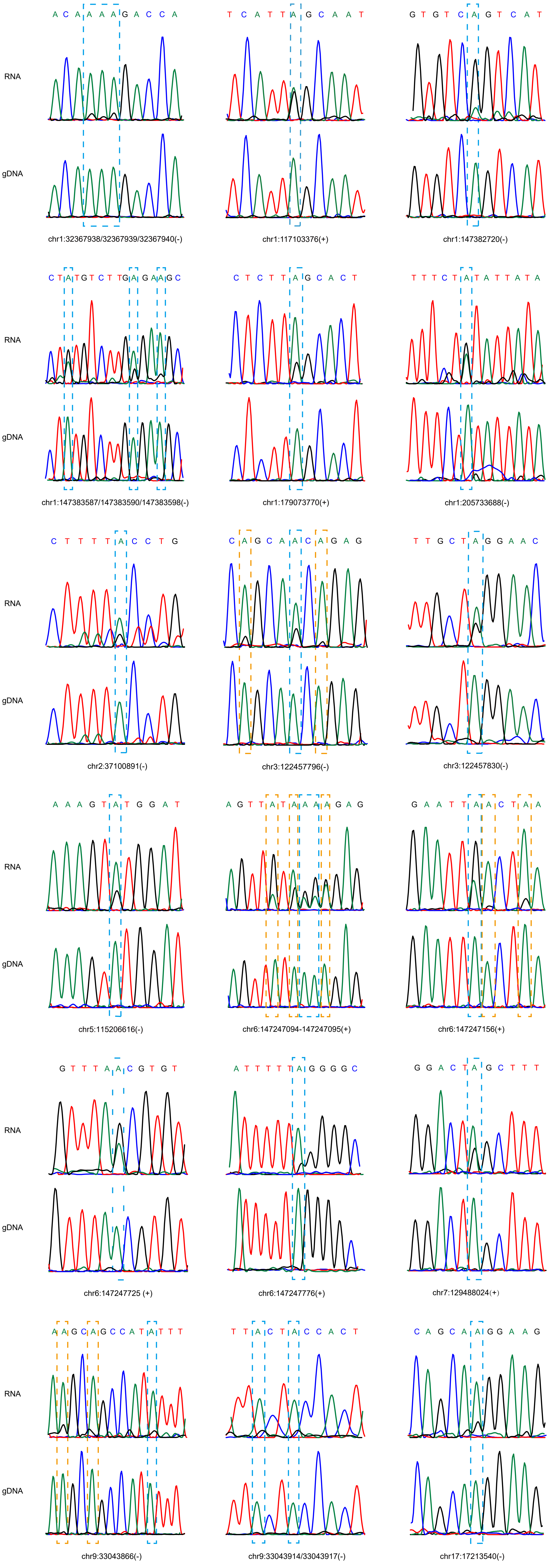

3 methods - unannotated

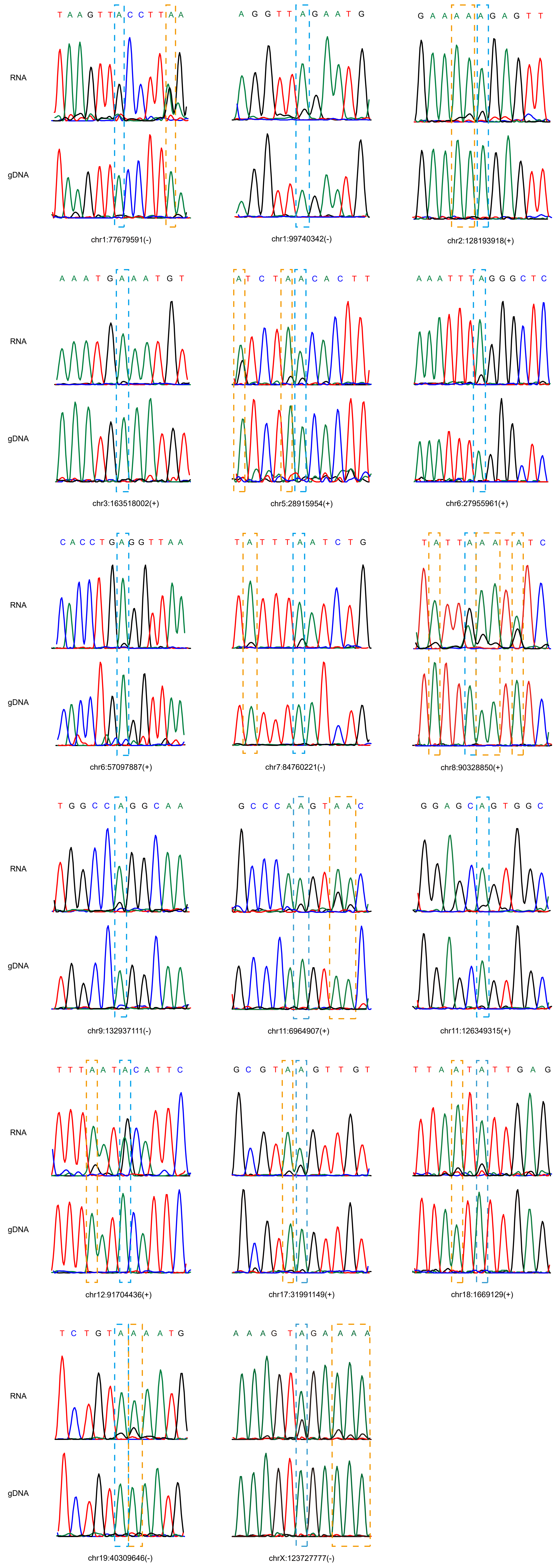

REDItools&RED-ML - annotated

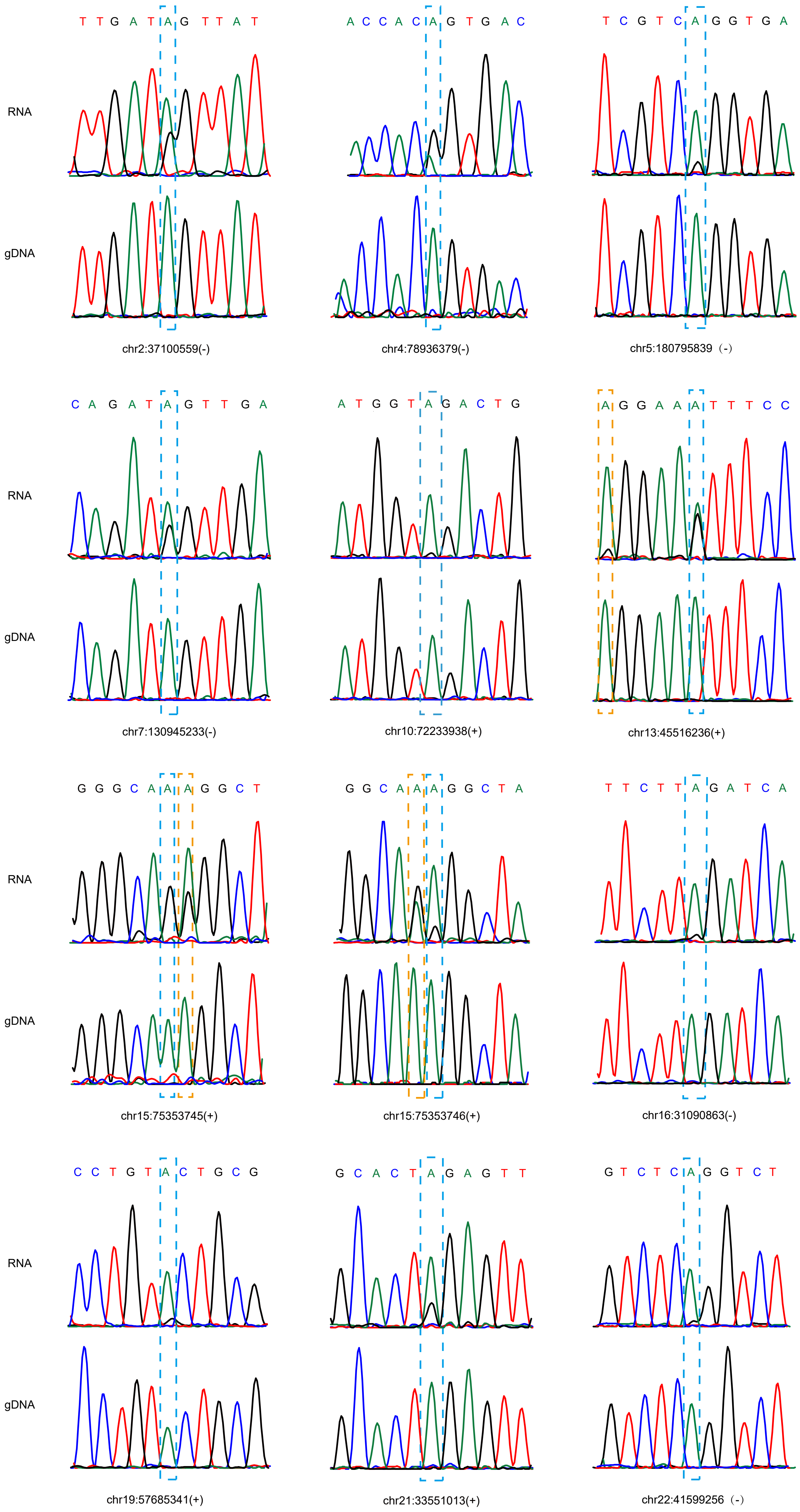

REDItools&RED-ML - unannotated

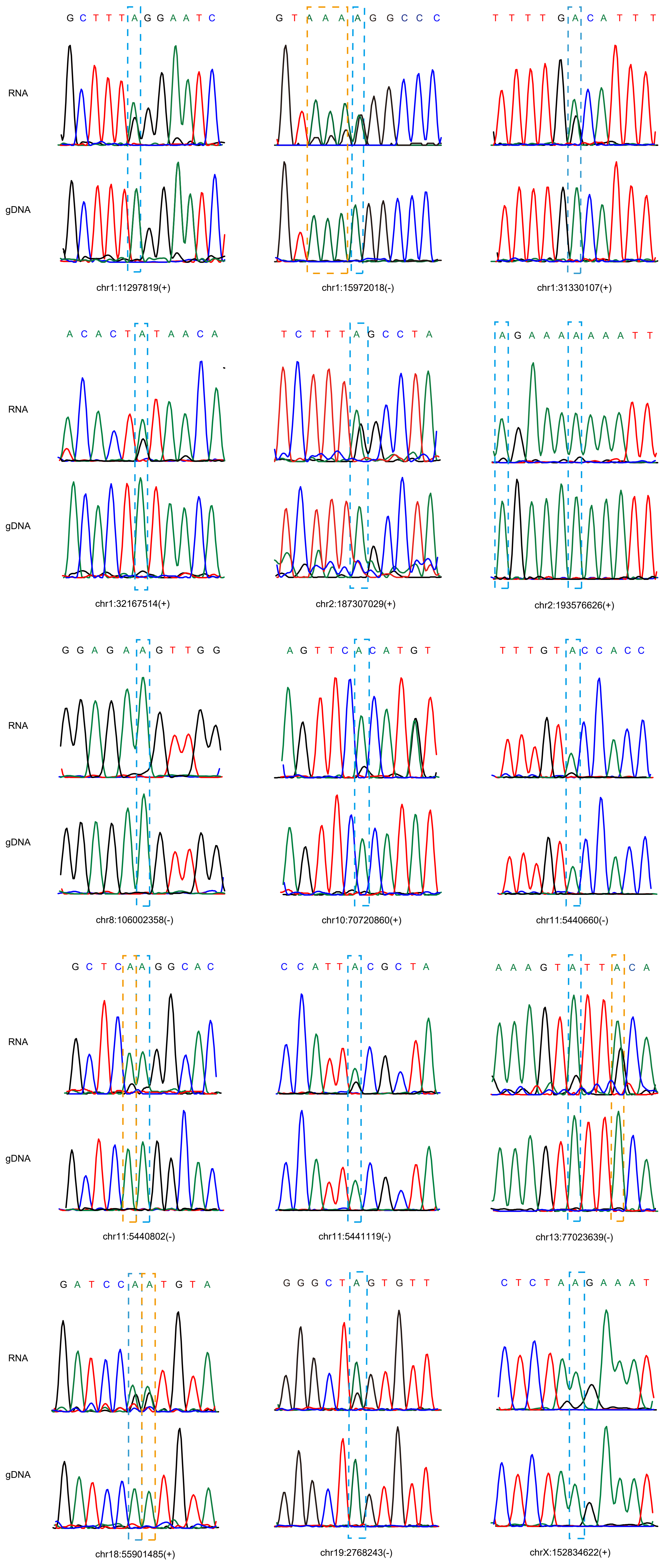

REDItools&SPRINT - annotated

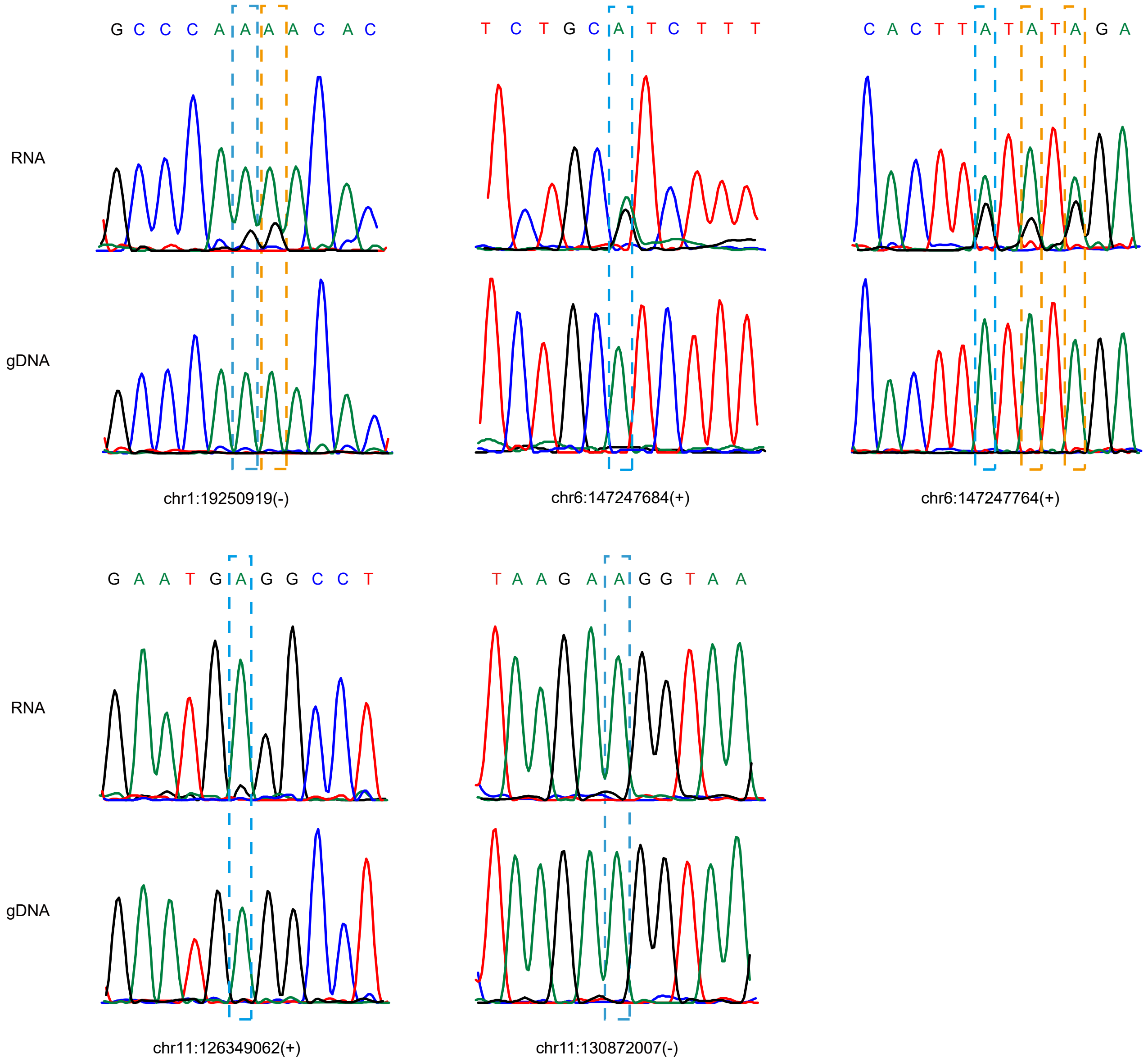

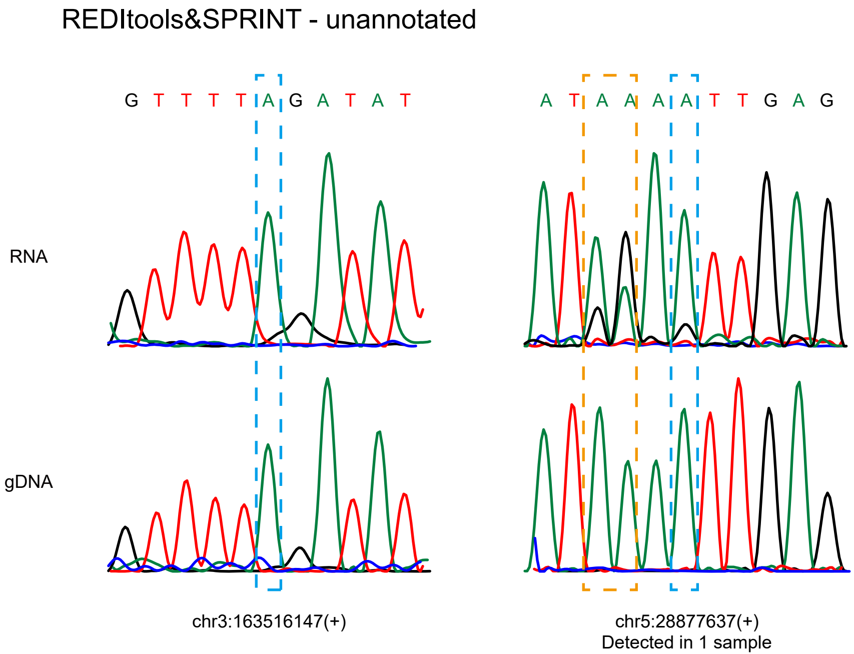
REDItools&SPRINT - unannotated
G T T T T A G A T A T
A T A A A A T T G A G
RNA
gDNA
chr3:163516147(+)
chr5:28877637(+)
Detected in 1 sample

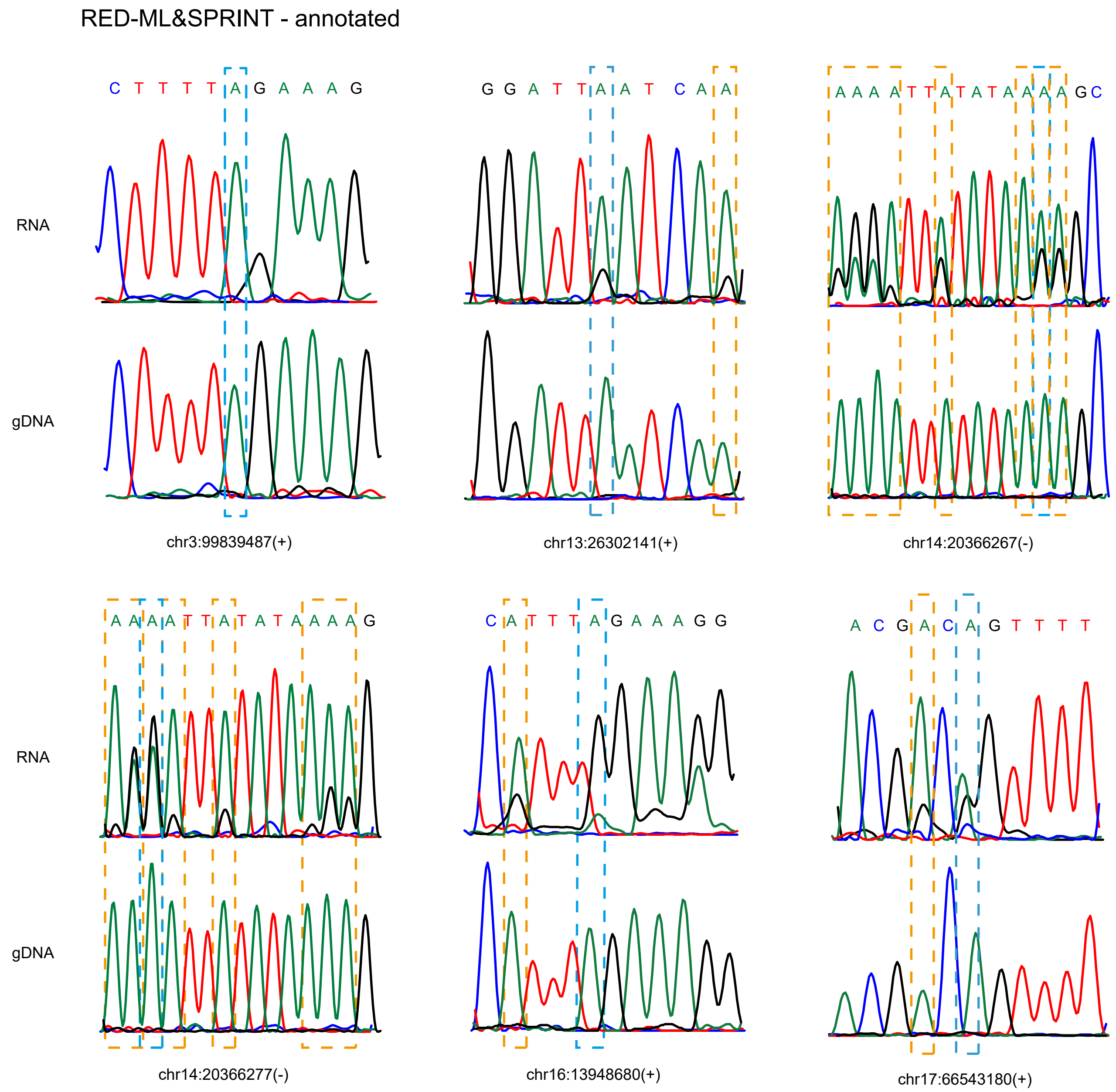
RED-ML&SPRINT - annotated
C T T T T A G A A A G
G G A T T A A T C A A
A A A A T T A T A T A A A A G C
RNA
gDNA
chr3:99839487(+)
chr13:26302141(+)
chr14:20366267(-)
A A A A T T A T A T A A A A G
C A T T T A G A A A G G
A C G A C A G T T T T
RNA
gDNA
chr14:20366277(-)
chr16:13948680(+)
chr17:66543180(+)

RED-ML&SPRINT - unannotated

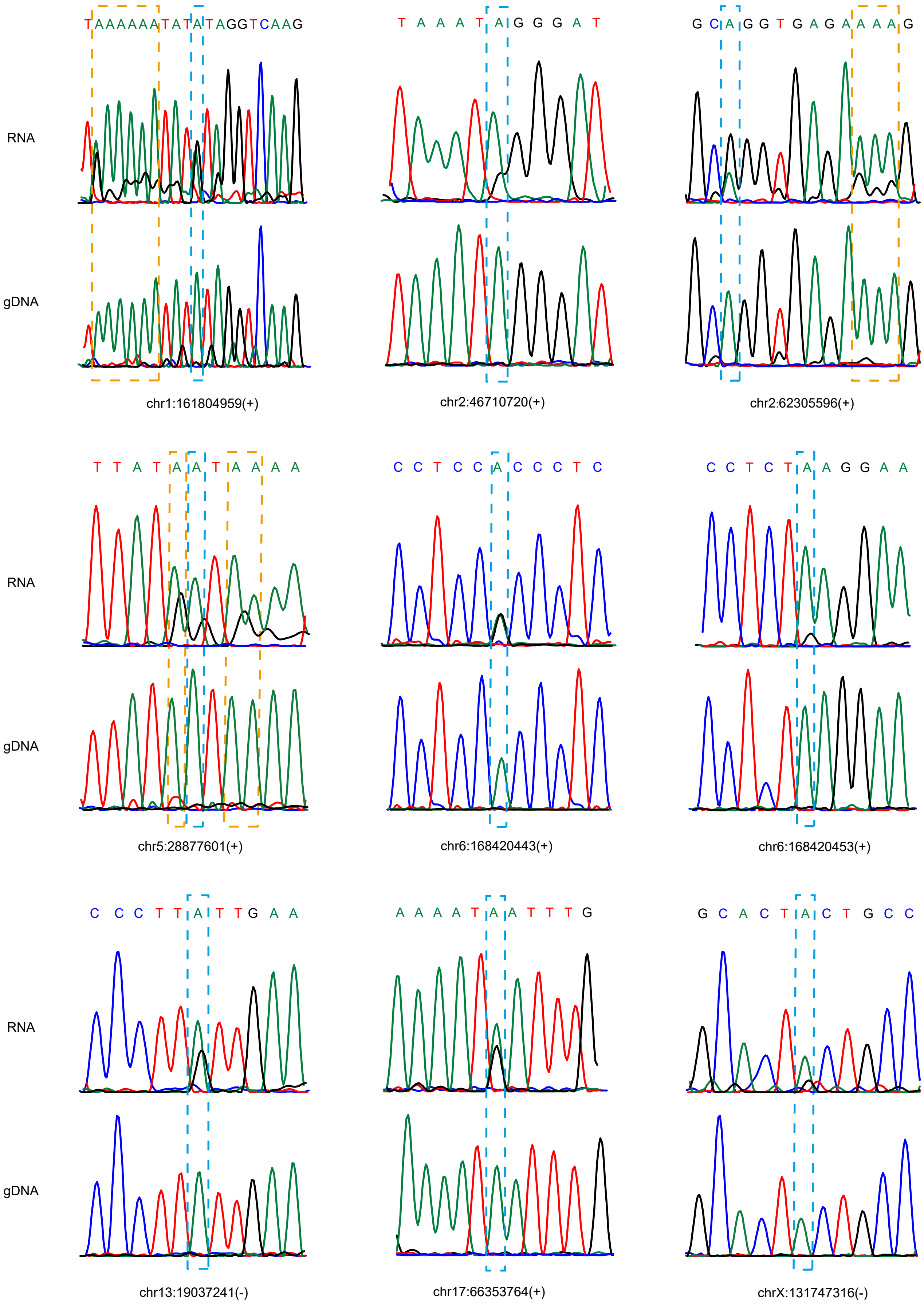

REDItools-specific - annotated

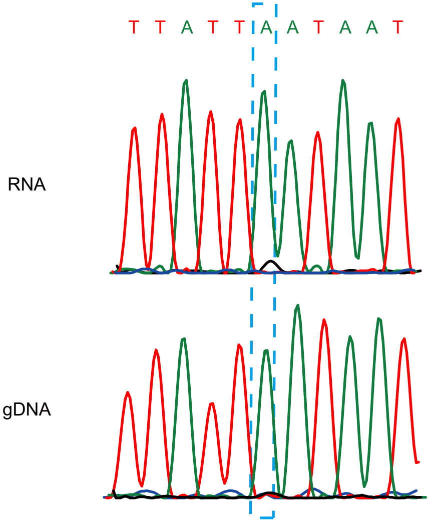

chr2:76193431(+)
Detected in 1 sample

REDItools-specific - unannotated

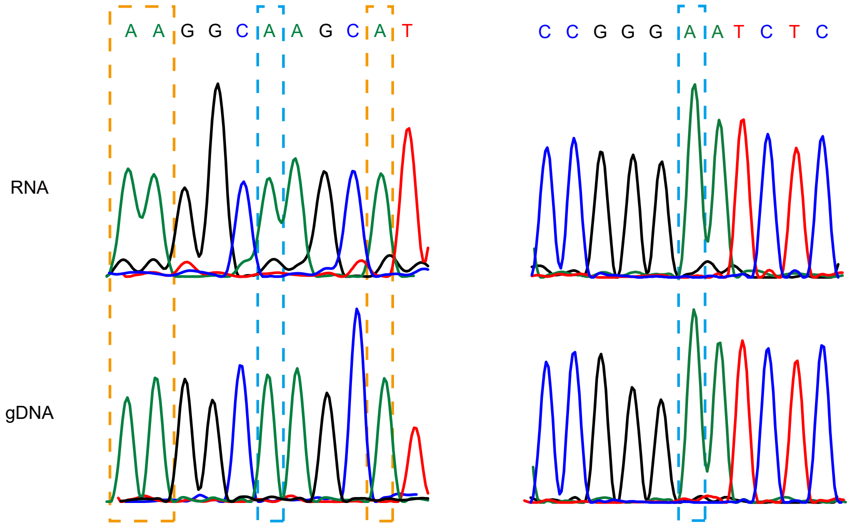

chr1:180198033 (+)
Detected in 1 sample

chr11:62665573(+)

RED-ML-specific - annotated

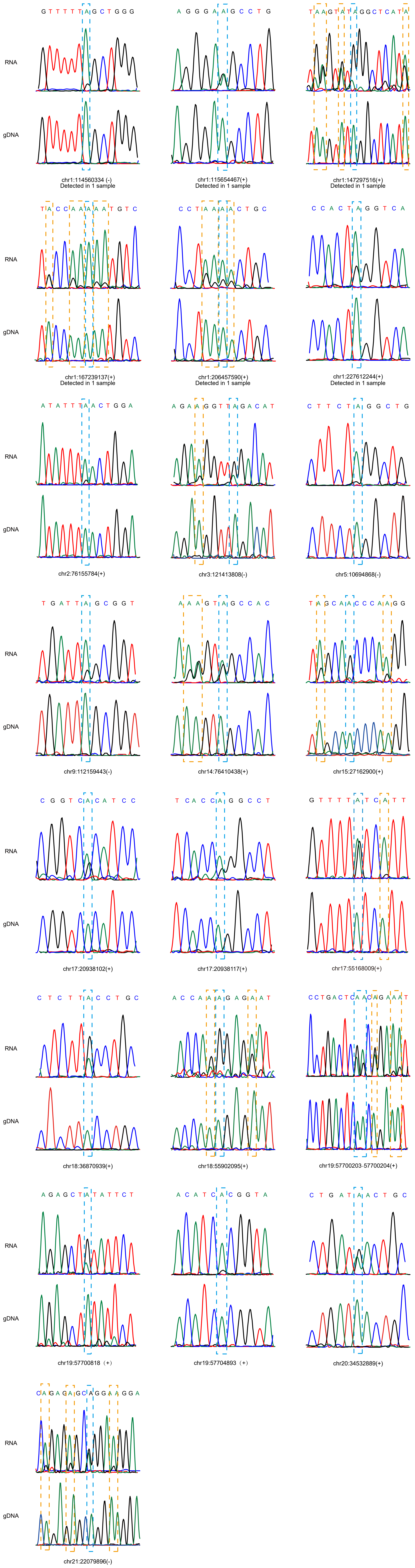

RED-ML-specific - unannotated

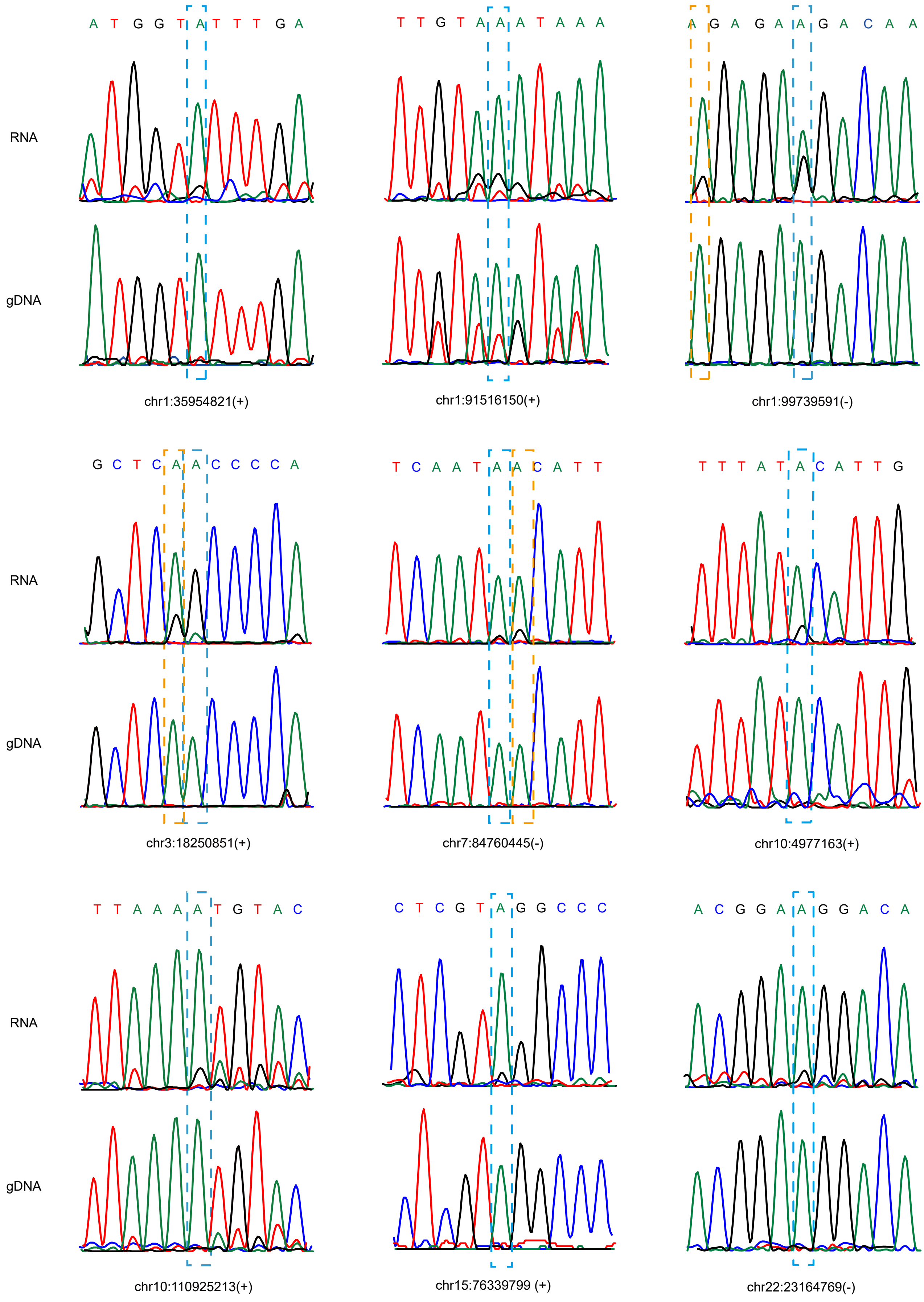

SPRINT-specific - annotated

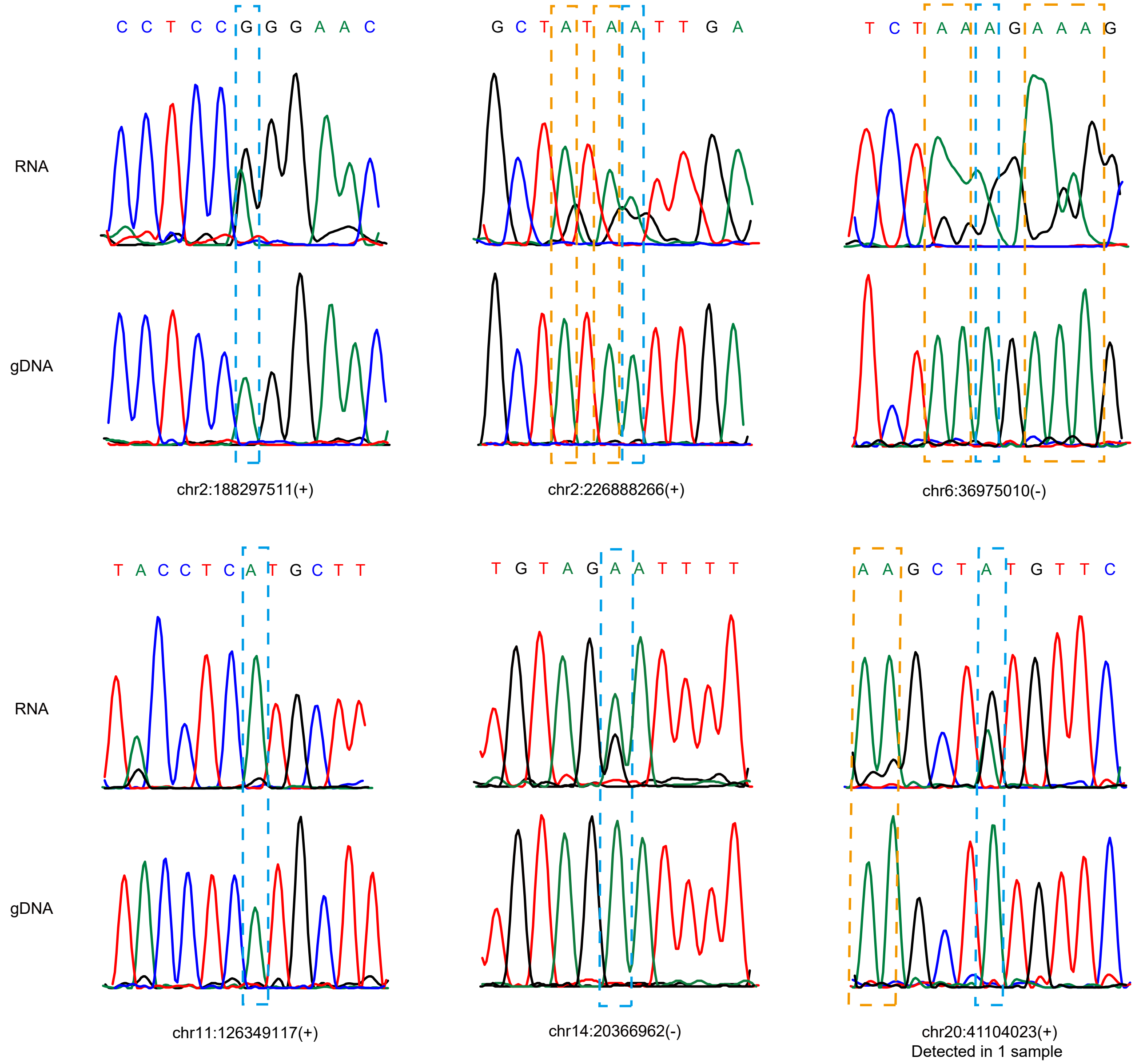

HPC-REDItools - annotated

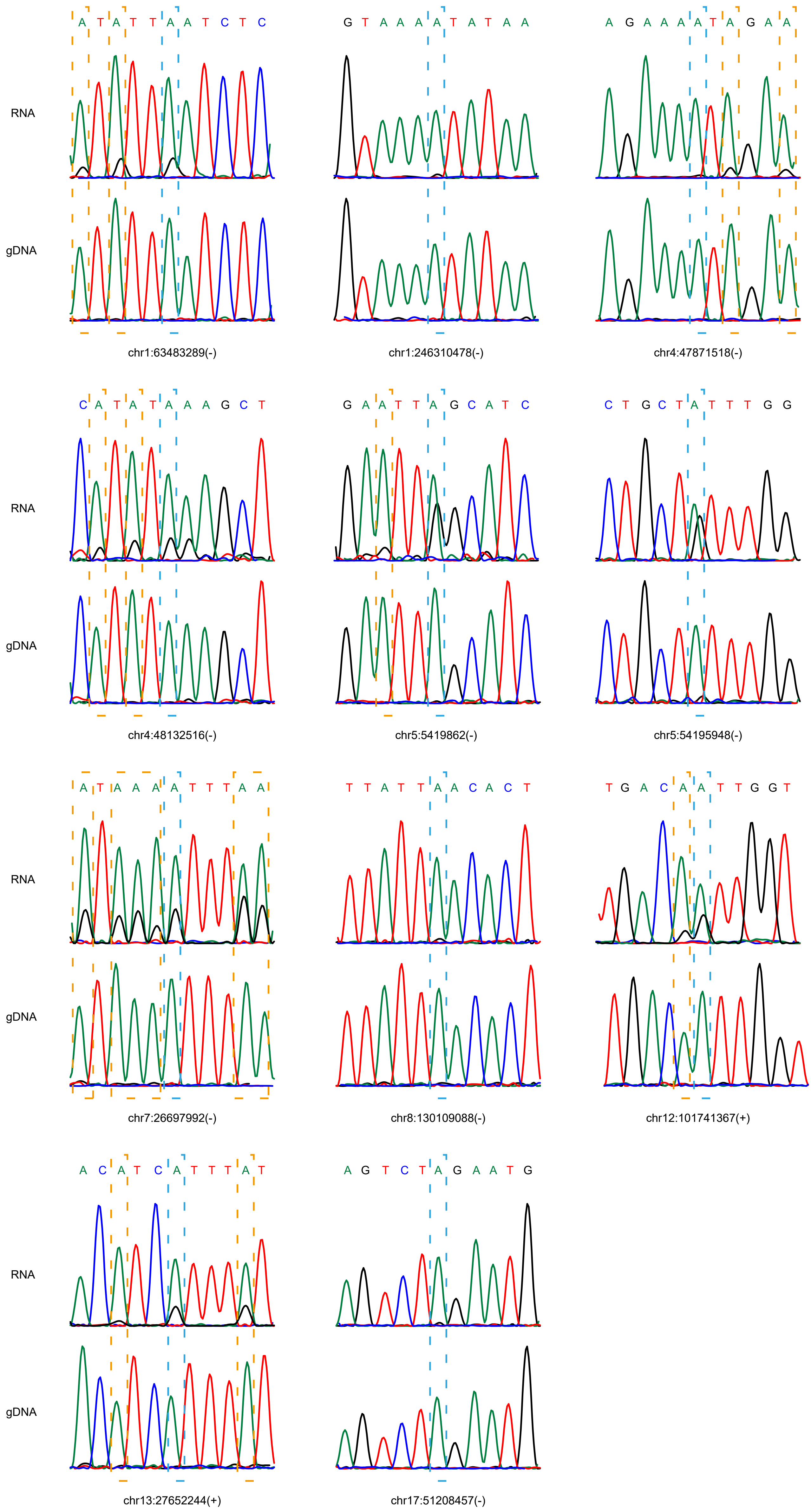

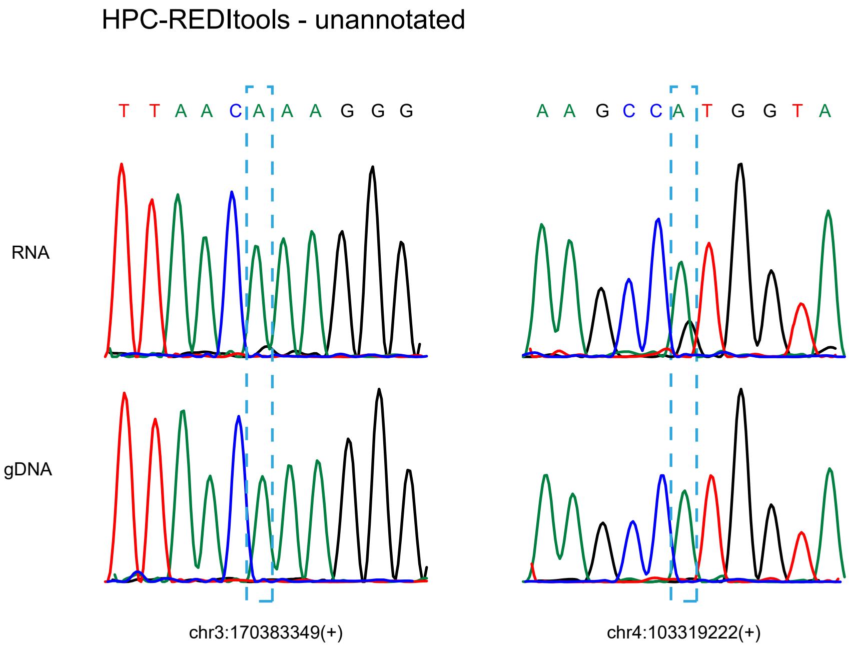
HPC-REDItools - unannotated
T T A A C A A A G G G
A A G C C A T G G T A
RNA
gDNA
chr3:170383349(+)
chr4:103319222(+)
