# Supplementary material for: Lessons from discovery of true ADAR RNA editing sites in a human cell line
Source: BMC Biol. 2023 Jul 19;21:160. doi: 10.1186/s12915-023-01651-w (PMC10357658; doi:10.1186/s12915-023-01651-w)

Additional file 5: Supplementary Figure 4. Sanger electropherograms of sites that were negative in the Sanger validation. Electropherograms for the target sites (blue dashed boxes) detected by each method or by multiple methods are shown for PCR products amplified from either (1) only RNA for the sites that do not show evidence of RNA editing, or (2) both RNA and genomic DNA (gDNA) for the sites that turned out to represent sequence variants. Adjacent editing sites identified by Sanger only are shown in yellow dashed boxes.

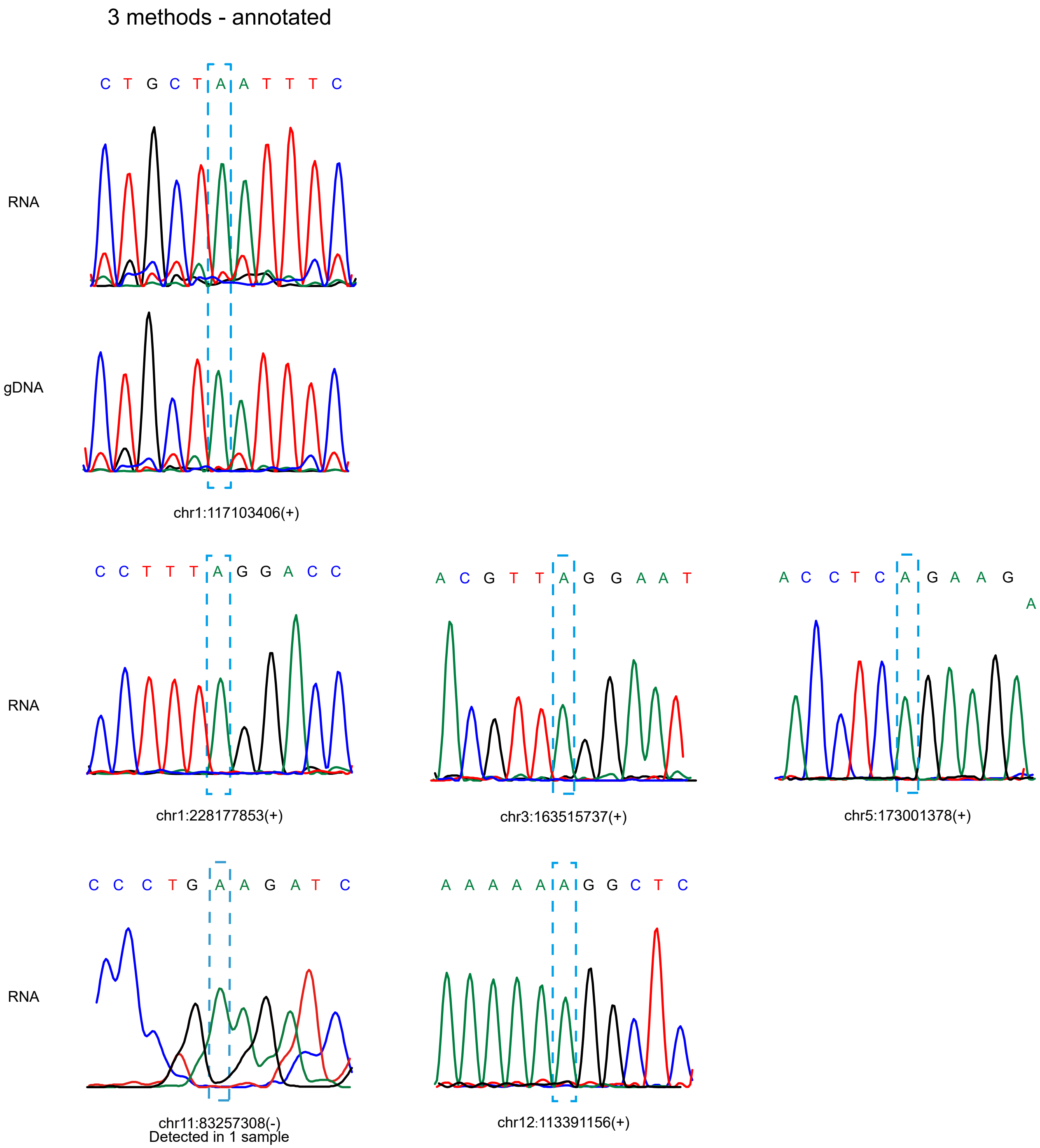

3 methods - unannotated

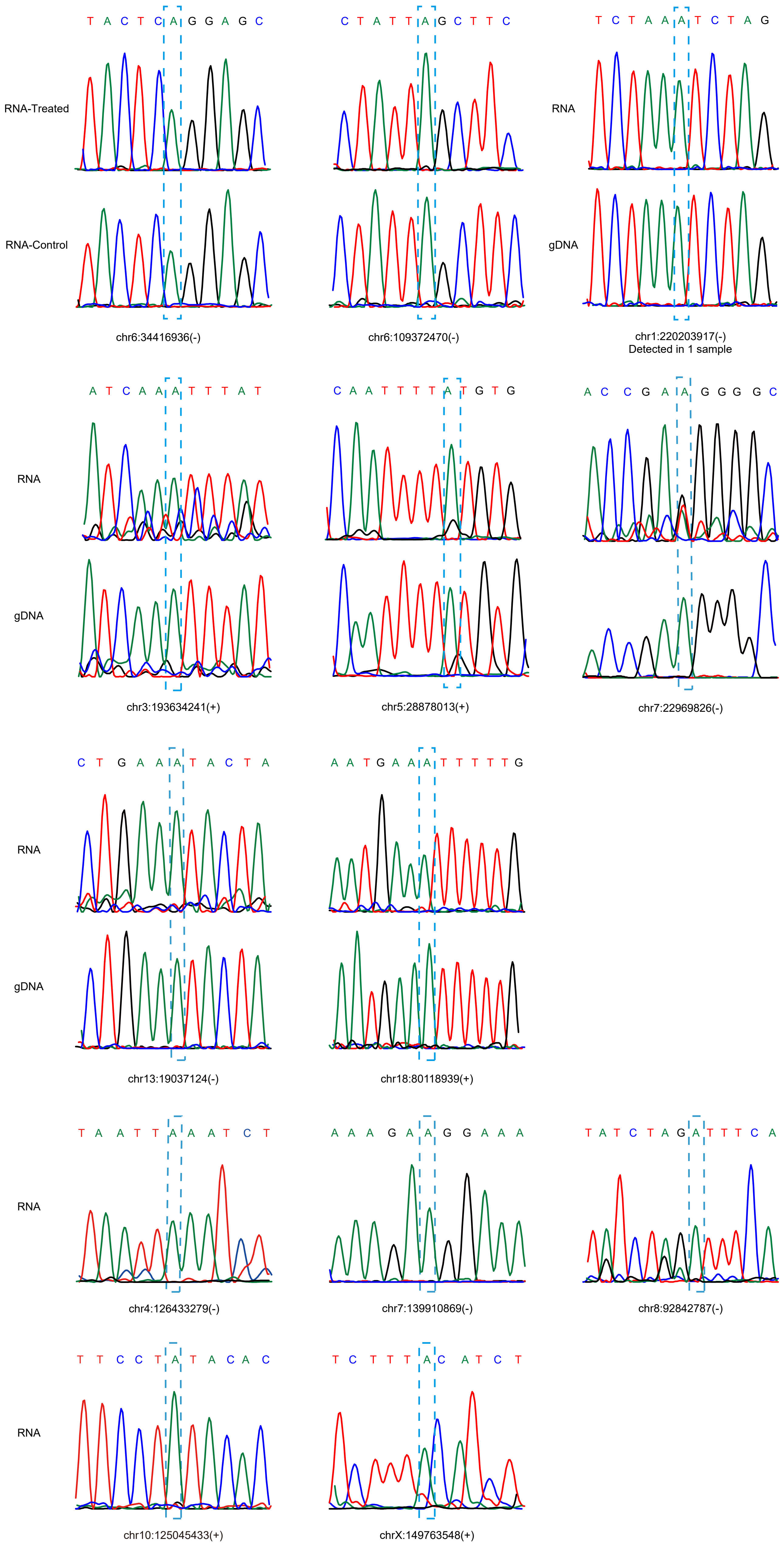

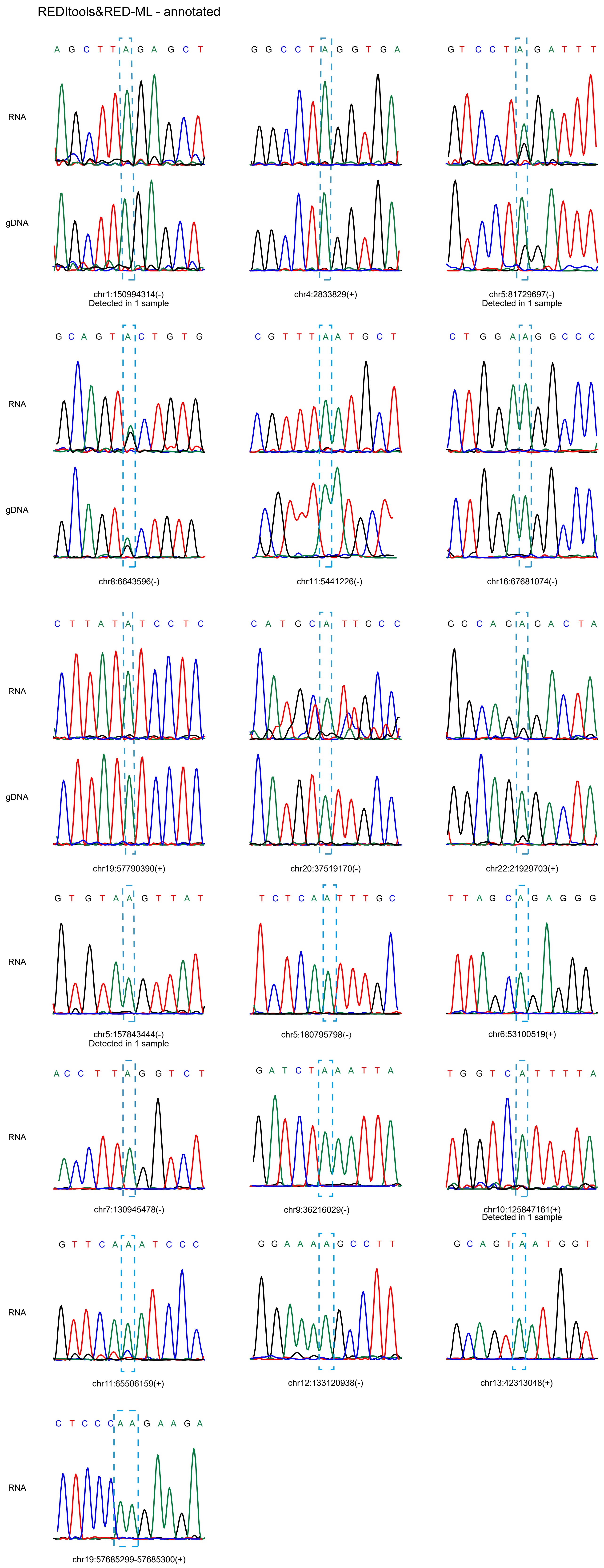
REDItools&RED-ML - annotated
A G C T T A G A G C T
G G C C T A G G T G A
G T C C T A G A T T T
RNA
gDNA
chr1:150994314(-)
Detected in 1 sample
chr4:2833829(+)
chr5:81729697(-)
Detected in 1 sample
G C A G T A C T G T G
C G T T T A A T G C T
C T G G A A G G C C C
RNA
gDNA
chr8:6643596(-)
chr11:5441226(-)
chr16:67681074(-)
C T T A T A T C C T C
C A T G C A T T G C C
G G C A G A G A C T A
RNA
gDNA
chr19:57790390(+)
chr20:37519170(-)
chr22:21929703(+)
G T G T A A G T T A T
T C T C A A T T T G C
T T A G C A G A G G G
RNA
chr5:157843444(-)
Detected in 1 sample
chr5:180795798(-)
chr6:53100519(+)
A C C T T A G G T C T
G A T C T A A A T T A
T G G T C A T T T T A
RNA
chr7:130945478(-)
chr9:36216029(-)
chr10:125847161(+)
Detected in 1 sample
G T T C A A A T C C C
G G A A A A G C C T T
G C A G T A A T G G T
RNA
chr11:65506159(+)
chr12:133120938(-)
chr13:42313048(+)
C T C C C A A G A A G A
RNA
chr19:57685299-57685300(+)

REDItools&RED-ML - unannotated

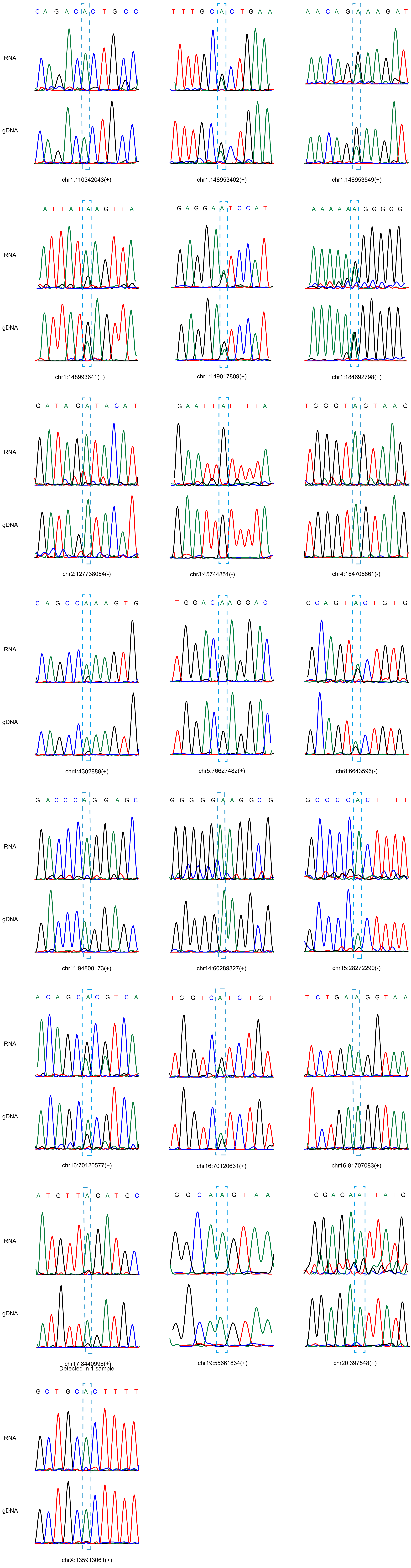

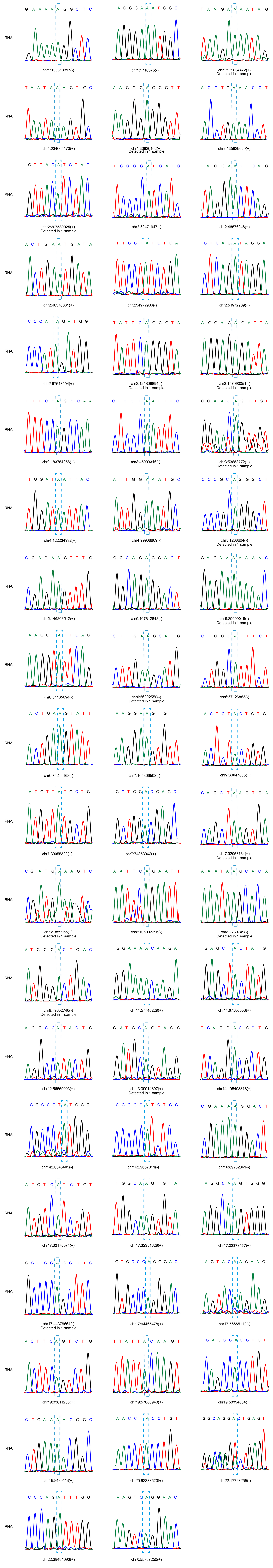
RNA
chr1:153813317(-)
chr1:1716375(-)
chr1:179634472(+)
Detected in 1 sample
chr1:234605173(+)
chr1:30936462(+)
Detected in 1 sample
chr2:135639020(+)
chr2:207580925(+)
Detected in 1 sample
chr2:32471947(-)
chr2:46576246(+)
chr2:46576601(+)
chr2:54972906(-)
chr2:54972909(+)
chr2:97648194(+)
chr3:121806894(-)
Detected in 1 sample
chr3:157090051(-)
Detected in 1 sample
chr3:183754258(+)
chr3:45003316(-)
chr3:53858772(+)
Detected in 1 sample
chr4:122234992(+)
chr4:99908889(-)
chr5:1268604(-)
Detected in 1 sample
chr5:146208512(+)
chr6:167842848(-)
chr6:29609016(-)
Detected in 1 sample
chr6:31165694(-)
chr6:56992550(-)
Detected in 1 sample
chr6:57126883(-)
chr6:75241168(-)
chr7:105306502(-)
chr7:30047886(+)
chr7:30055322(+)
chr7:74353962(+)
chr7:92058764(+)
Detected in 1 sample
chr8:1859965(+)
Detected in 1 sample
chr8:106002296(-)
chr8:2739749(-)
Detected in 1 sample
chr9:79652740(-)
Detected in 1 sample
chr11:57740229(+)
chr11:67586653(+)
chr12:56569003(+)
chr13:39014397(+)
Detected in 1 sample
chr14:105498818(+)
chr14:20343409(-)
chr16:29667011(-)
chr16:89282361(-)
chr17:32175971(+)
chr17:32351629(+)
chr17:32373457(+)
chr17:44378664(-)
Detected in 1 sample
chr17:64465478(+)
chr17:76685112(-)
chr19:33811253(+)
chr19:57686943(+)
chr19:58394804(+)
chr19:8489113(+)
chr20:62388520(+)
chr22:17728255(-)
chr22:38484093(+)
chrX:55757250(+)

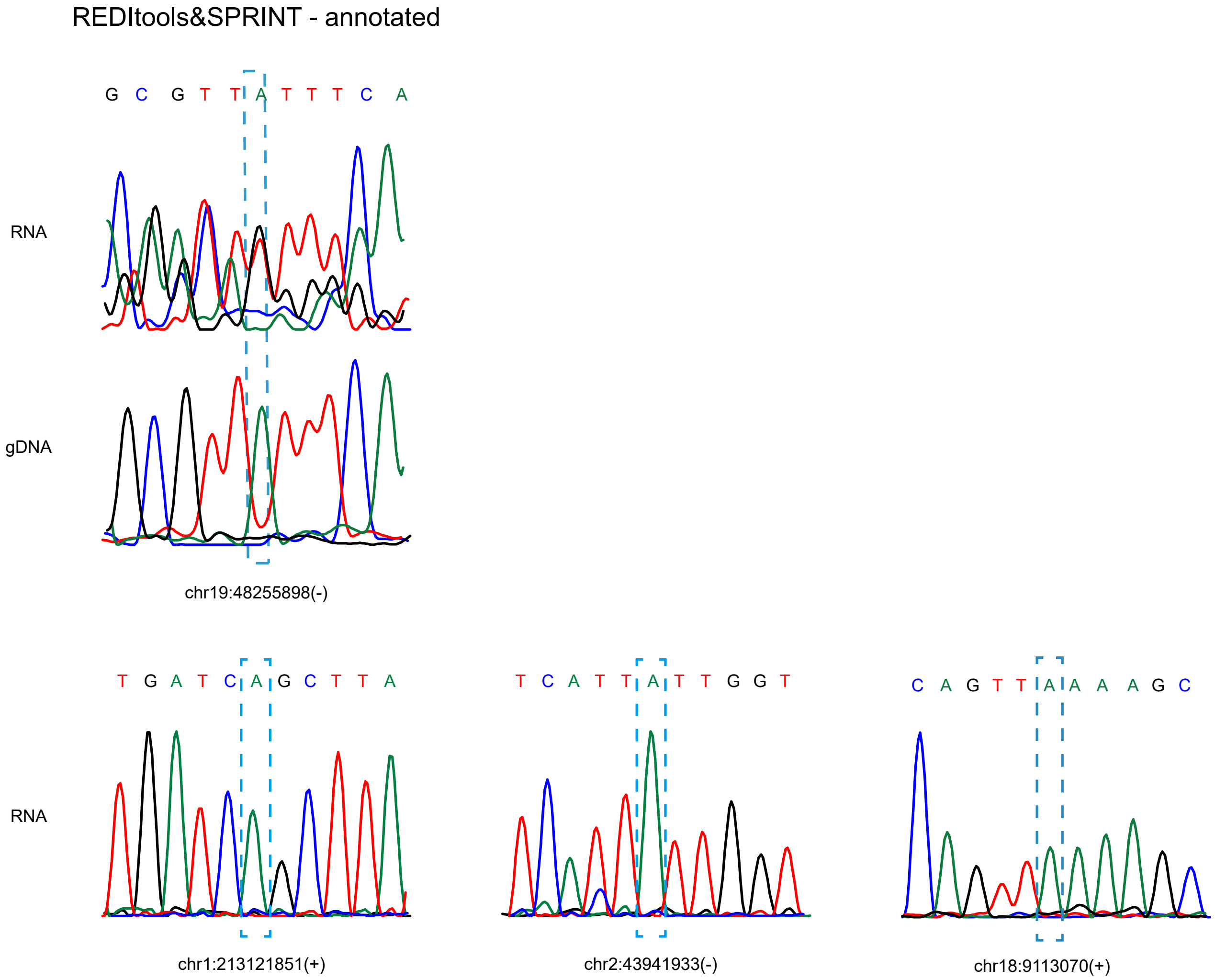
REDItools&SPRINT - annotated
G C G T T A T T T C A
RNA
gDNA
chr19:48255898(-)
T G A T C A G C T T A
RNA
chr1:213121851(+)
T C A T T A T T G G T
chr2:43941933(-)
C A G T T A A A A G C
chr18:9113070(+)

REDItools&SPRINT - unannotated

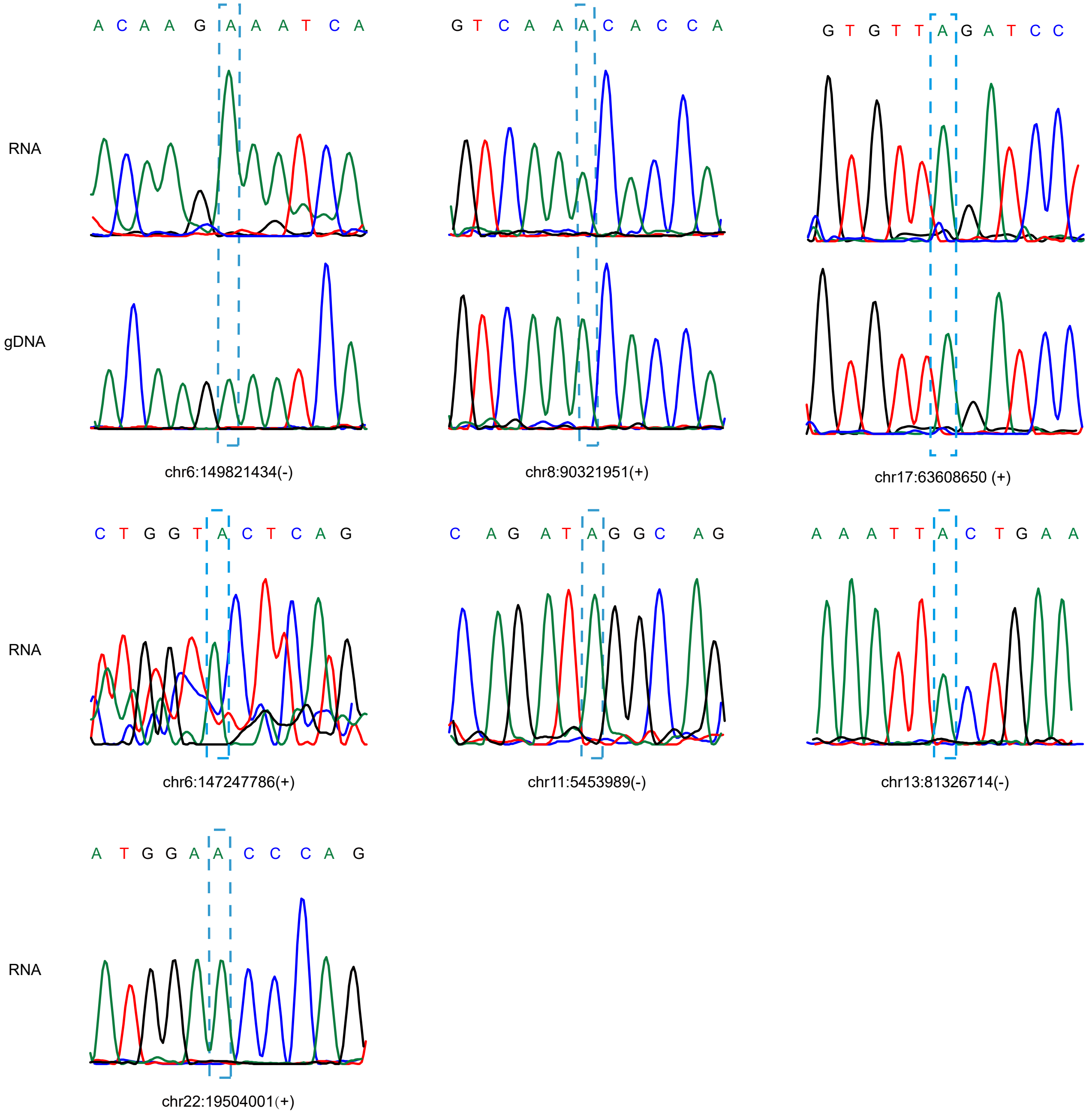

RED-ML&SPRINT - annotated

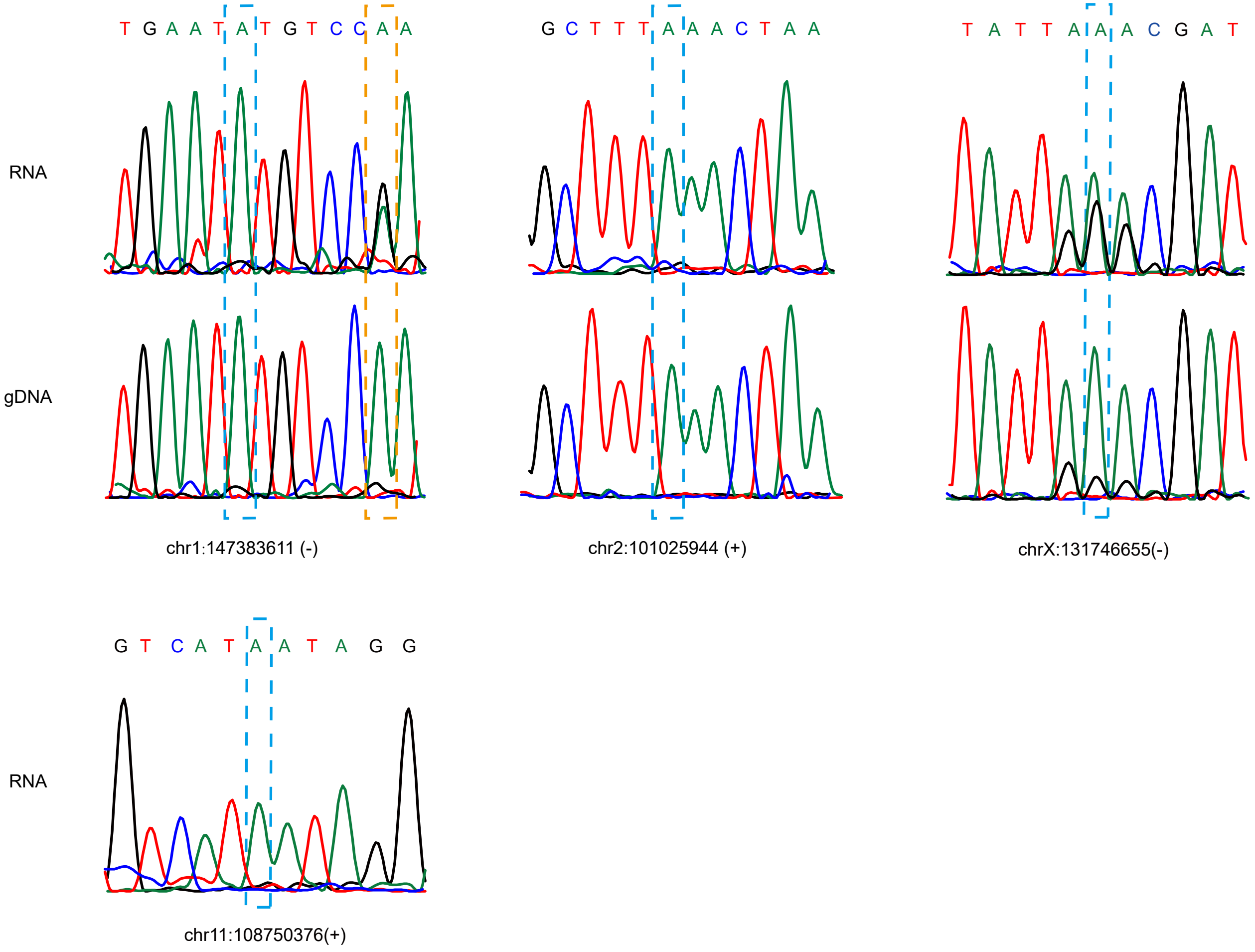

RED-ML&SPRINT - unannotated

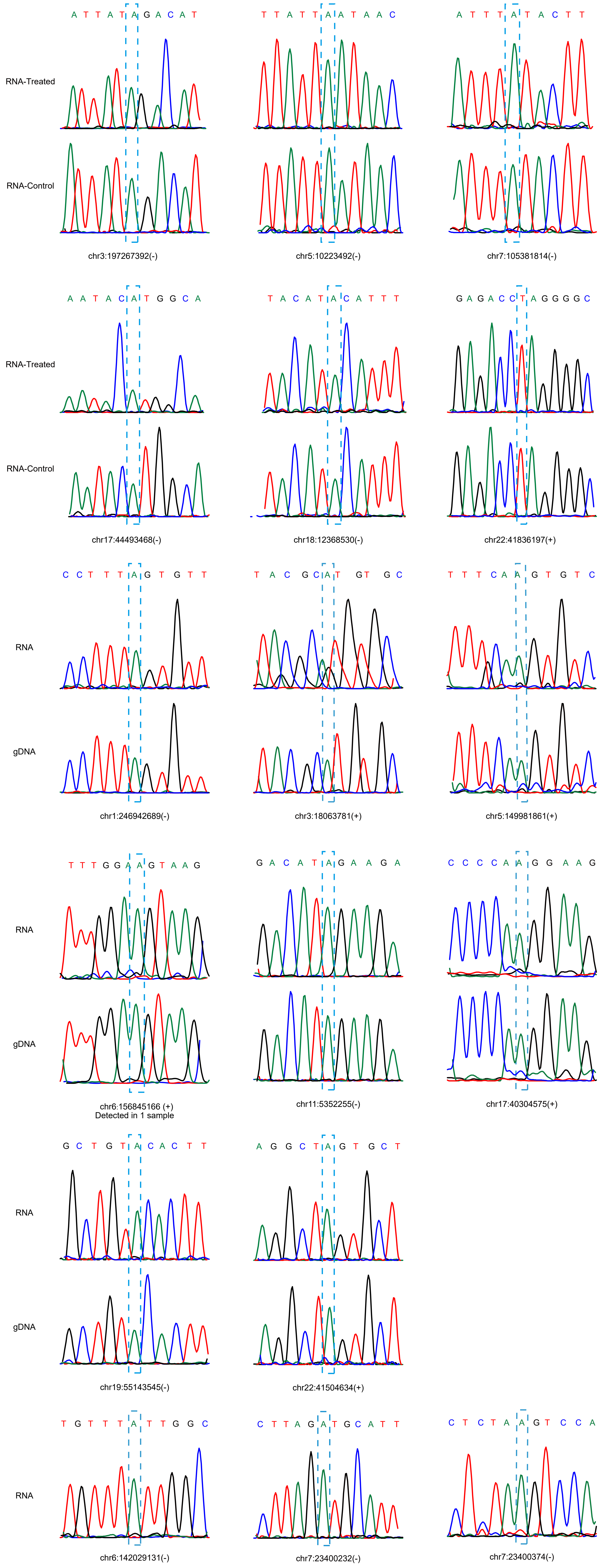

REDItools-specific - annoatated

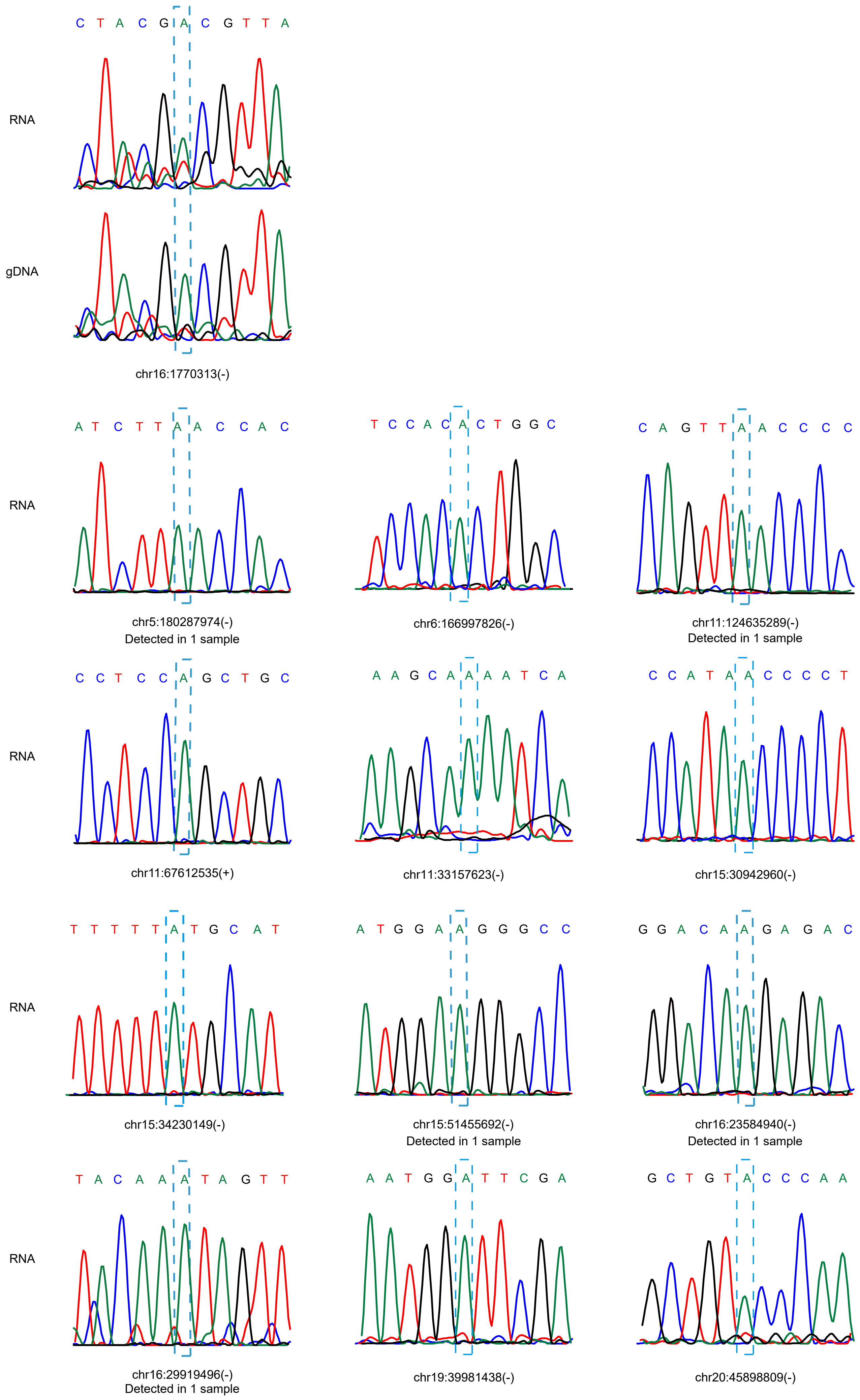

REDItools-specific - unannotated

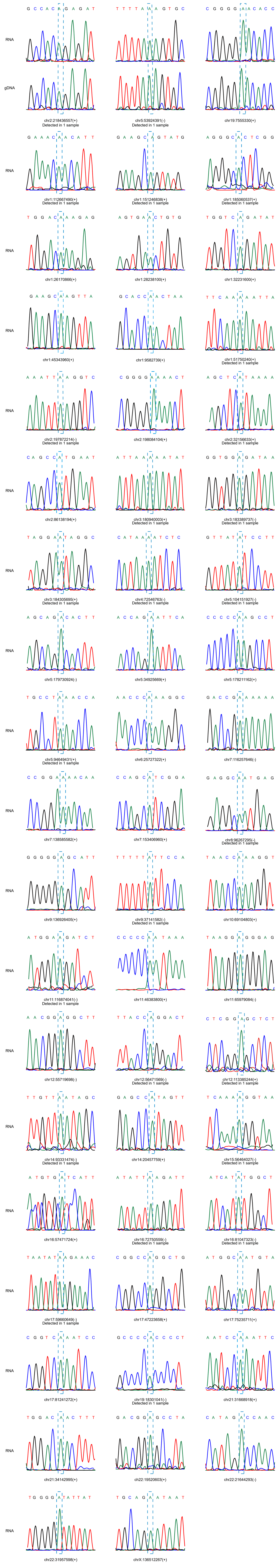

RED-ML-specific - annotated

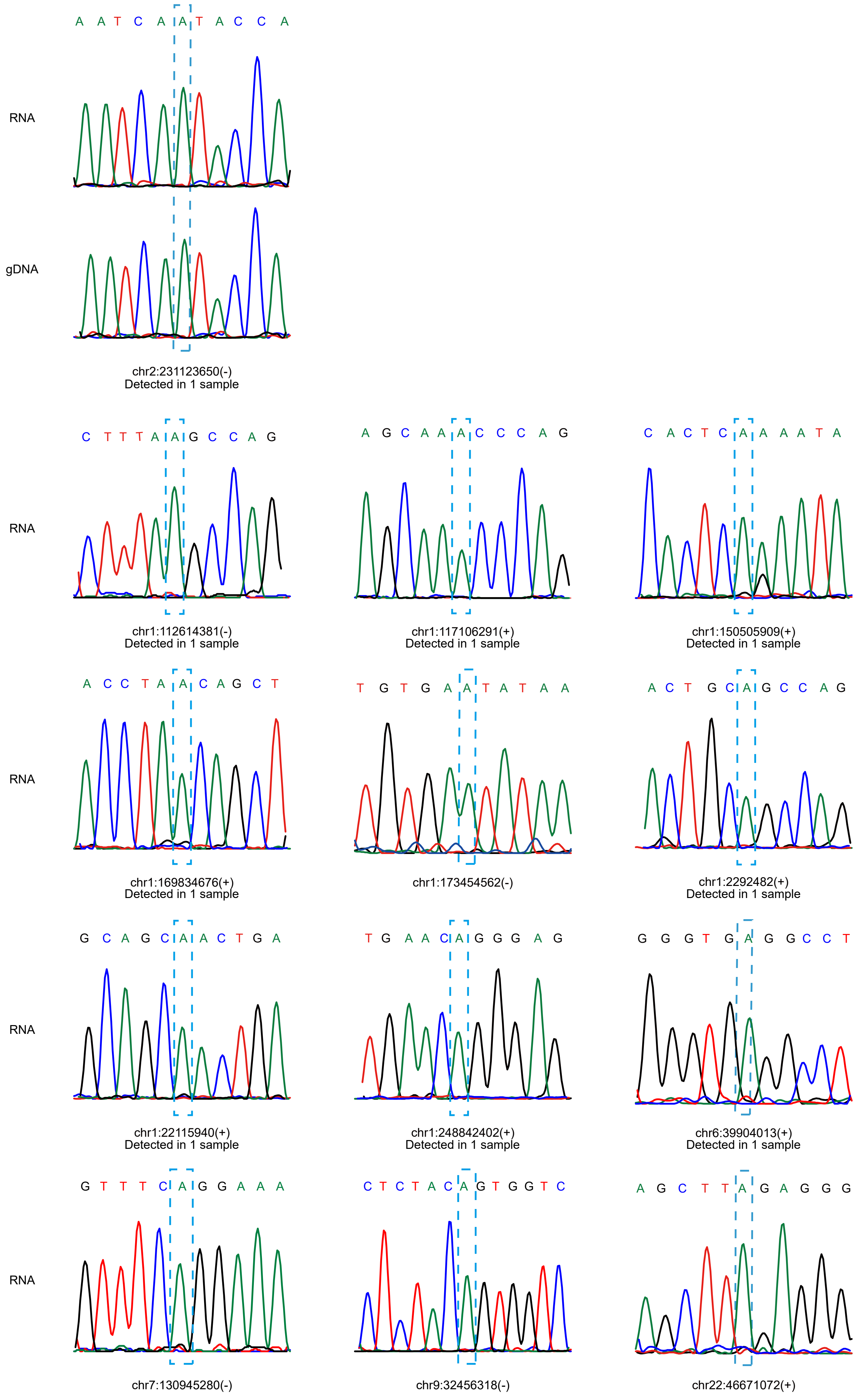

RED-ML-specific - unannotated

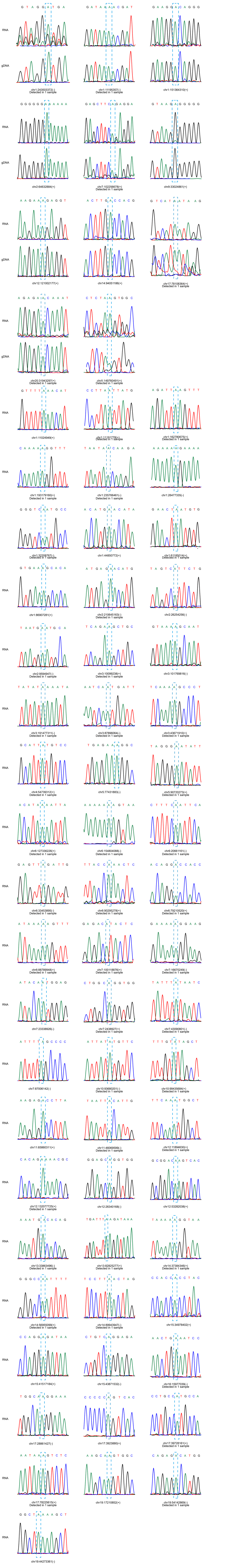

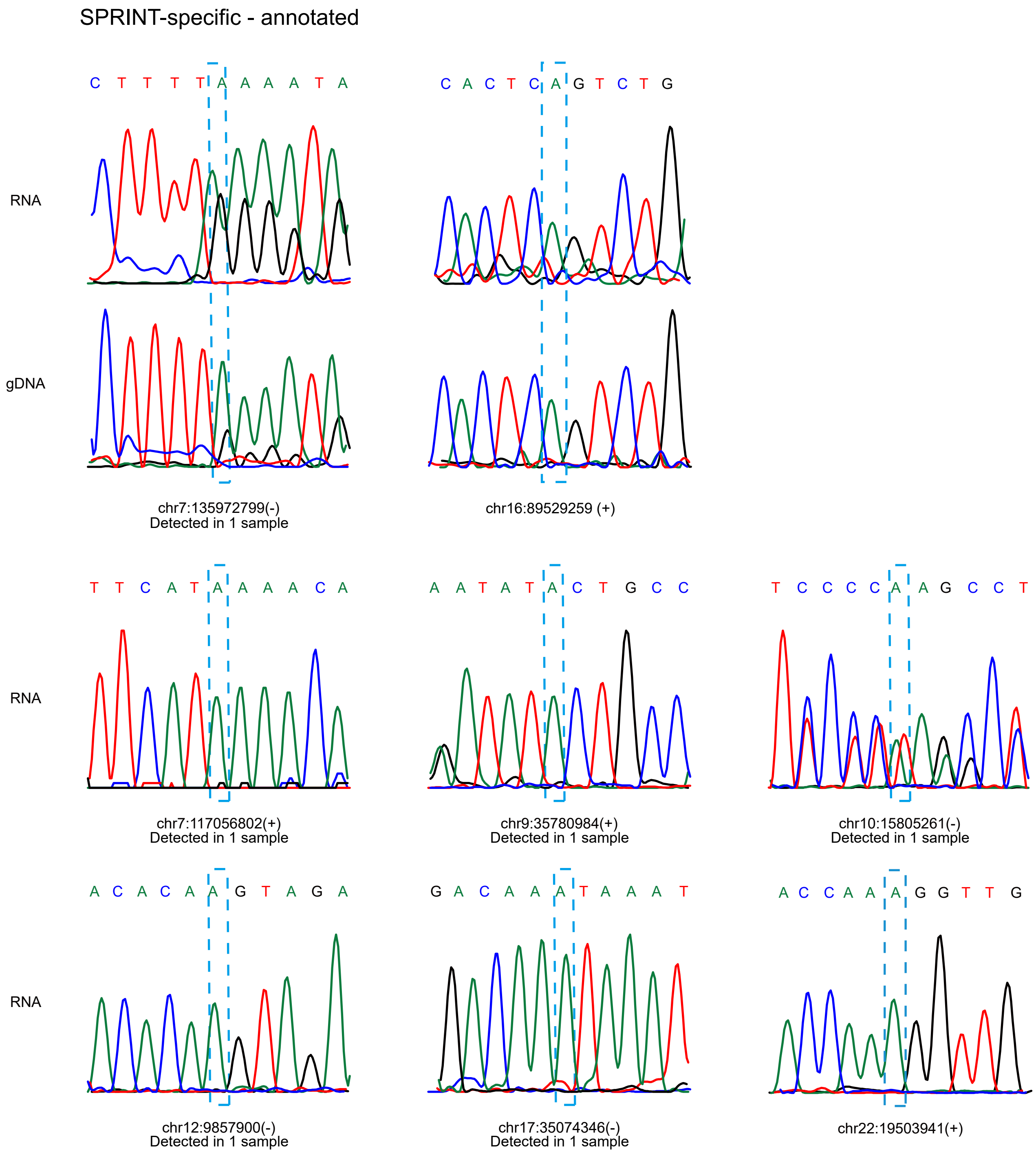
SPRINT-specific - annotated
C T T T T A A A A T A
C A C T C A G T C T G
RNA
gDNA
chr7:135972799(-)
Detected in 1 sample
chr16:89529259 (+)
T T C A T A A A A C A
A A T A T A C T G C C
T C C C C A A G C C T
RNA
chr7:117056802(+)
Detected in 1 sample
chr9:35780984(+)
Detected in 1 sample
chr10:15805261(-)
Detected in 1 sample
A C A C A A G T A G A
G A C A A A T A A A T
A C C A A A G G T T G
RNA
chr12:9857900(-)
Detected in 1 sample
chr17:35074346(-)
Detected in 1 sample
chr22:19503941(+)

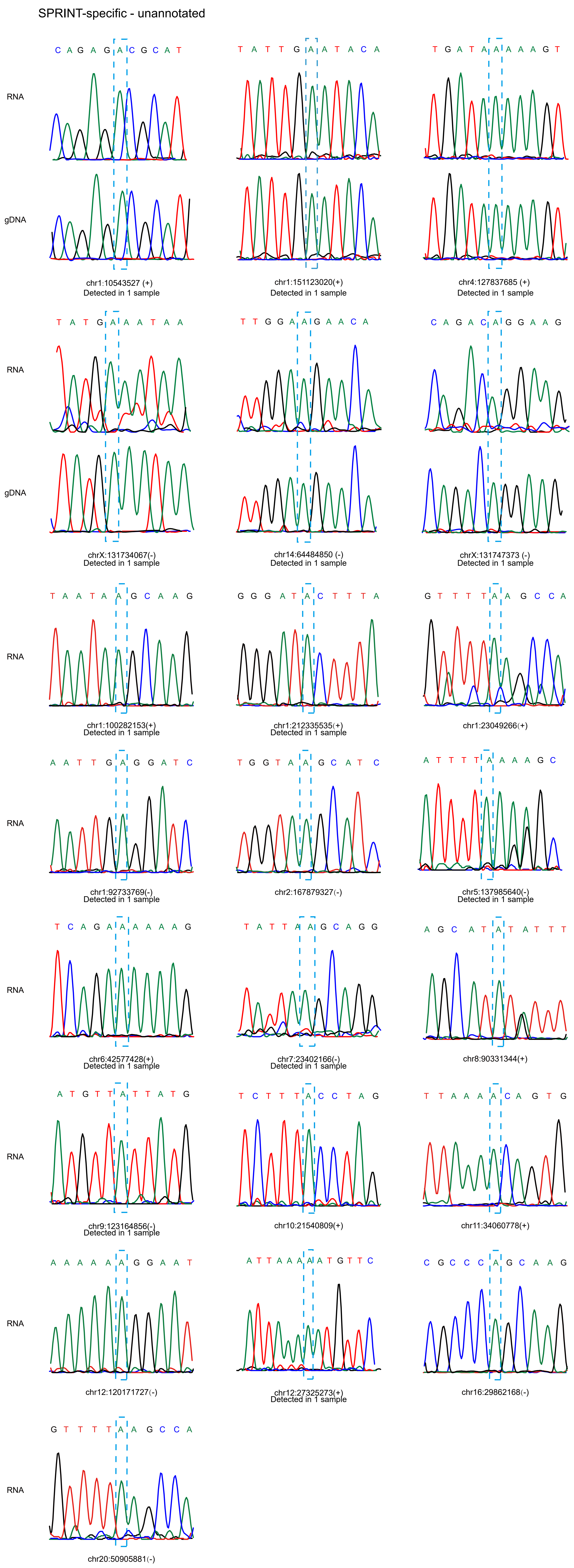
SPRINT-specific - unannotated
RNA
gDNA
chr1:10543527 (+)
Detected in 1 sample
chr1:151123020(+)
Detected in 1 sample
chr4:127837685 (+)
Detected in 1 sample
chrX:131734067(-)
Detected in 1 sample
chr14:64484850 (-)
Detected in 1 sample
chrX:131747373 (-)
Detected in 1 sample
chr1:100282153(+)
Detected in 1 sample
chr1:212335535(+)
Detected in 1 sample
chr1:23049266(+)
chr1:92733769(-)
Detected in 1 sample
chr2:167879327(-)
chr5:137985640(-)
Detected in 1 sample
chr6:42577428(+)
Detected in 1 sample
chr7:23402166(-)
Detected in 1 sample
chr8:90331344(+)
chr9:123164856(-)
Detected in 1 sample
chr10:21540809(+)
chr11:34060778(+)
chr12:120171727(-)
chr12:27325273(+)
Detected in 1 sample
chr16:29862168(-)
chr20:50905881(-)

HPC-REDItools - annotated

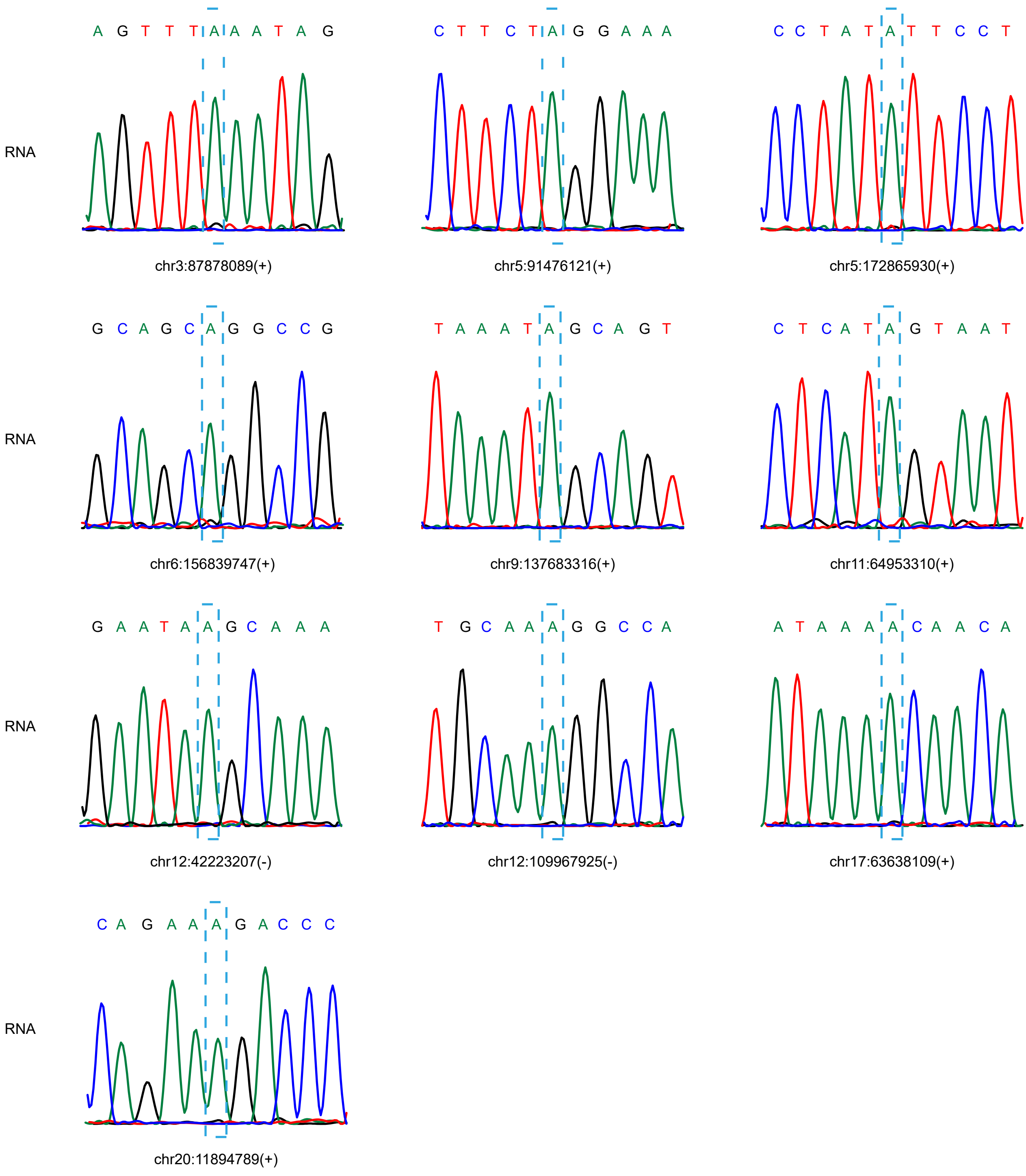

HPC-REDItools - unannotated

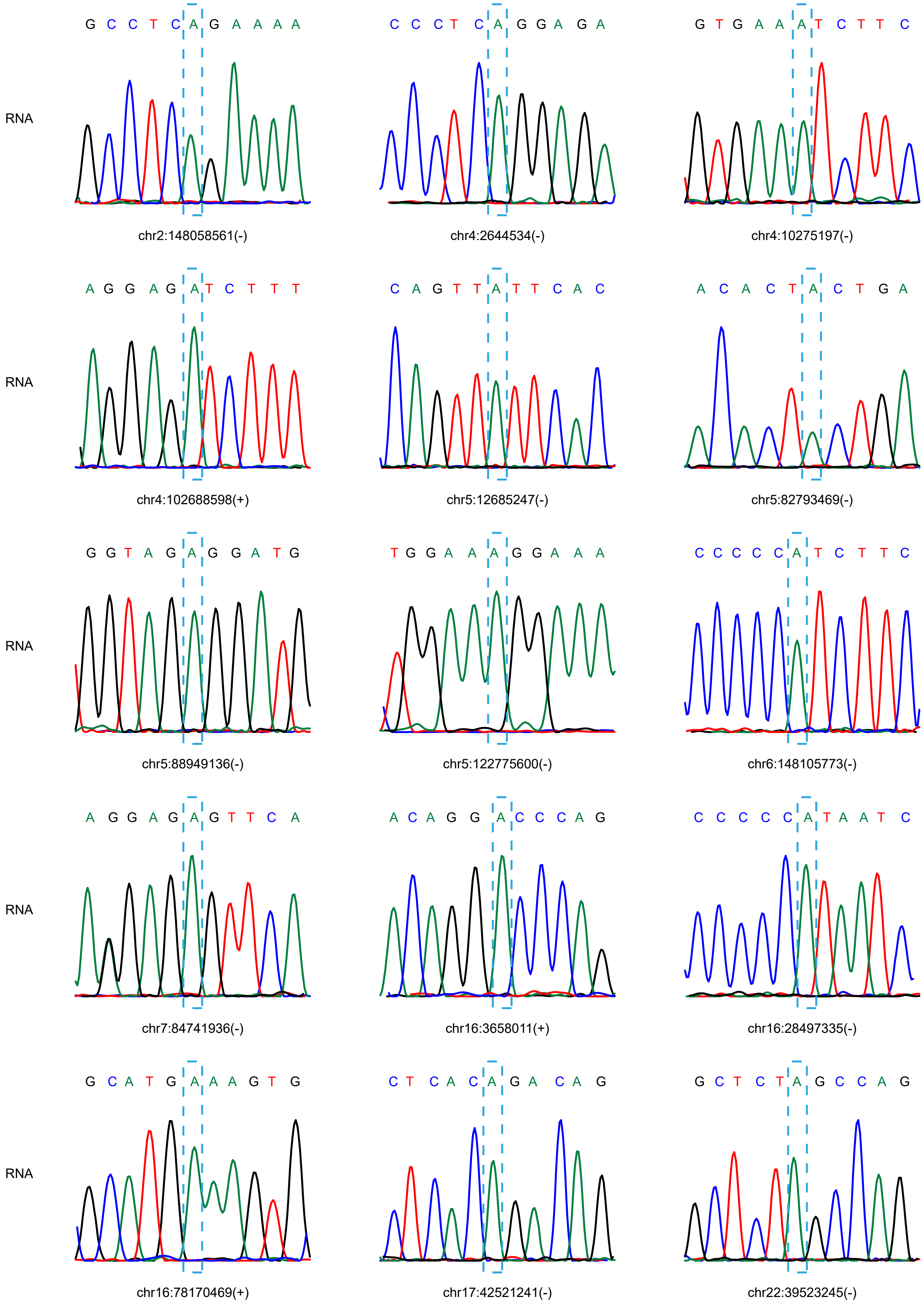

Supplement: Supplementary file 5 — Additional file 5: SupplementaryFigure 4. Sanger electropherograms of sites that were negativein the Sanger validation. Electropherograms for the target sites (blue dashedboxes) detected by each method or by multiple methods are shown for PCR productsamplified from either (1) only RNA for the sites that did not show evidence ofRNA editing, or (2) both RNA and genomic DNA (gDNA) for the sites that turnedout to represent sequence variants. Adjacent editing sites identified by Sangeronly are shown in yellow dashed boxes. [file 12915_2023_1651_MOESM5_ESM.pdf]
