# Supplementary material for: Lessons from discovery of true ADAR RNA editing sites in a human cell line
Source: BMC Biol. 2023 Jul 19;21:160. doi: 10.1186/s12915-023-01651-w (PMC10357658; doi:10.1186/s12915-023-01651-w)
Supplement: Supplementary file 6 — Additional file 6: SupplementaryFigure 5. Comparison of HPC-REDItools and the 3 methods(REDItools, RED-ML, SPRINT) used in our pipeline. Venn diagrams representingthe number of annotated (a) and unannotated (b) sites which were detected byone or more different methods in at least 2 samples. (c) Validation ratios(Y-axis) of the annotated (left) and unannotated (right) sites detected byREDItools only (grey), HPC-REDItools only (dark blue) and the pipeline (red).The values in the bars represent the total number of corresponding sites.Source data are provided as a Source data file. [file 12915_2023_1651_MOESM6_ESM.pdf]

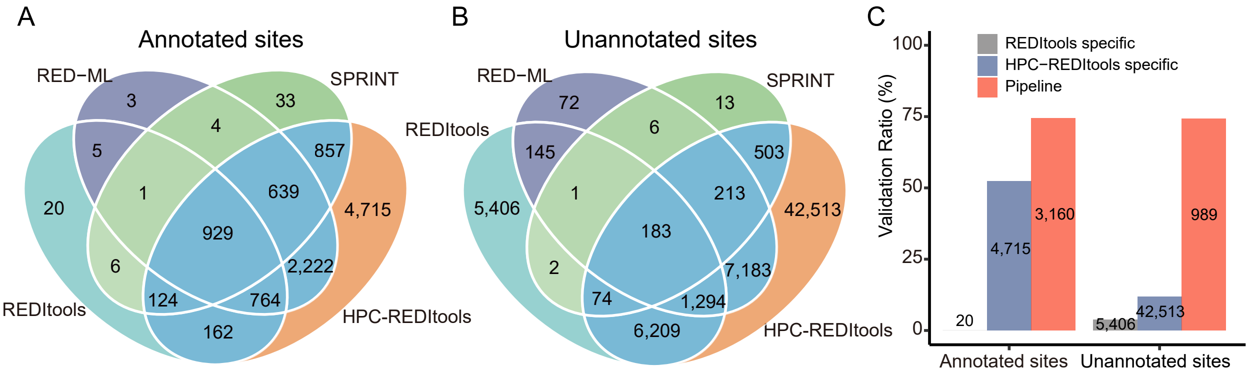

Additional file 6: Supplementary Figure 5. Comparison of HPC-REDItools and the 3 methods (REDItools, RED-ML, SPRINT) used in our pipeline. Venn diagrams representing the number of annotated (a) and unannotated (b) sites which were detected by one or more different methods in at least 2 samples. (c) Validation ratios (Y-axis) of the annotated (left) and unannotated (right) sites detected by REDItools only (grey), HPC-REDItools only (dark blue) and the pipeline (red). The values in the bars represent the total number of corresponding sites. Source data are provided as a Source data file.
