# Supplementary material for: Lessons from discovery of true ADAR RNA editing sites in a human cell line
Source: BMC Biol. 2023 Jul 19;21:160. doi: 10.1186/s12915-023-01651-w (PMC10357658; doi:10.1186/s12915-023-01651-w)
Supplement: Supplementary file 7 — Additional file 7: SupplementaryFigure 6. Distribution of transcript abundance and editinglevel of all sites subjected to Sanger validation. (a) Read depths in the lnscale and (b) editing levels of sites that were positive (left) and negative(right) in the Sanger validation. The red dashed lines represent the numbers ofreads or editing levels for each quartile. The percentages on the right representthe validation ratios of sites which have read depths or editing levels thatare higher than those represented by the red dashed lines. Source data areprovided as a Source data file. [file 12915_2023_1651_MOESM7_ESM.pdf]

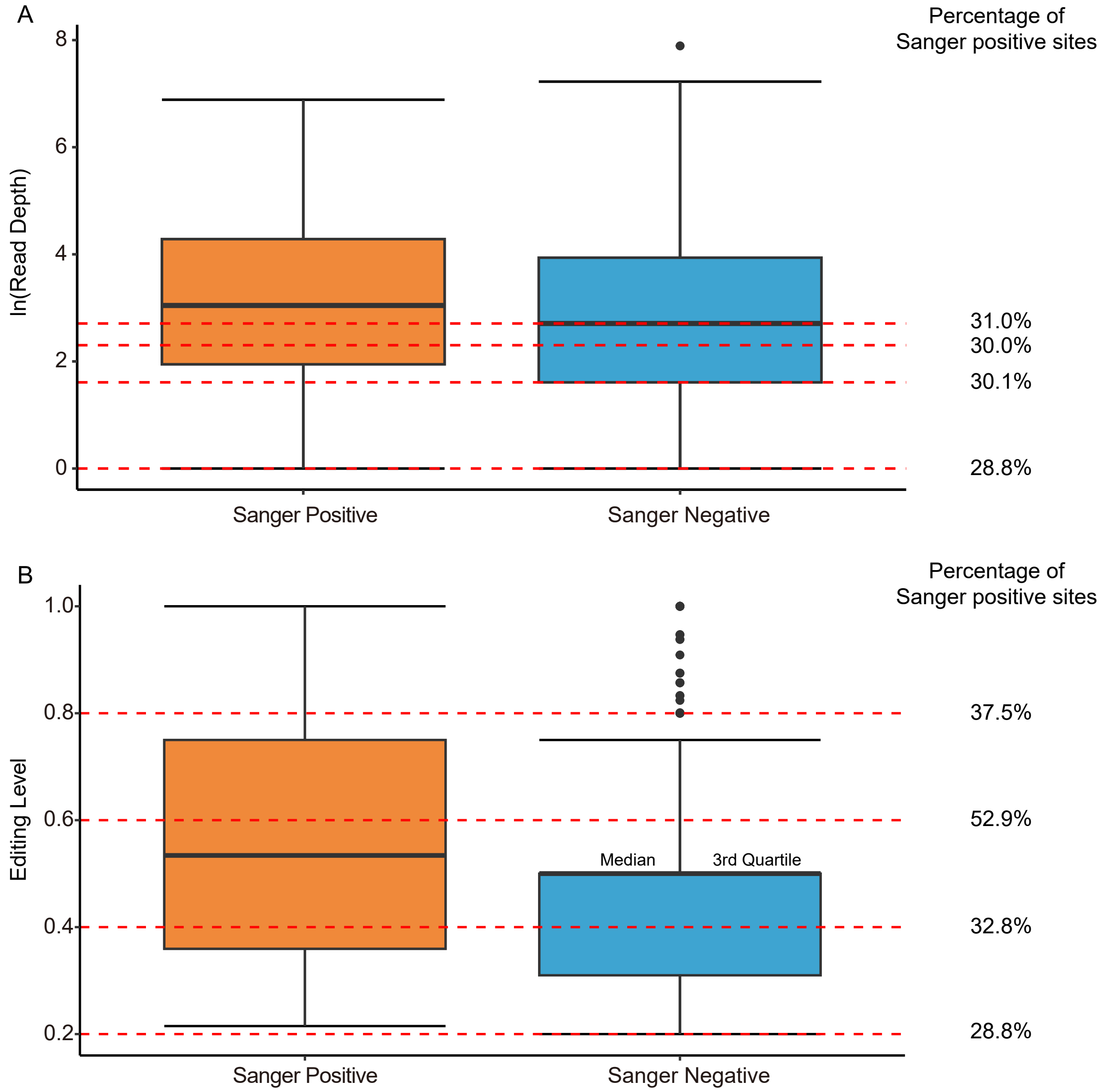

Additional file 7: Supplementary Figure 6. Distribution of transcript abundance and editing level of all sites subjected to Sanger validation. (a) Read depths in the ln scale and (b) editing levels of sites that were positive (left) and negative (right) in the Sanger validation. The red dashed lines represent the numbers of reads or editing levels for each quartile. The percentages on the right represent the validation ratios of sites which have read depths or editing levels that are higher than those represented by the red dashed lines. Source data are provided as a Source data file.
